# Radiation effects on the mixed convection flow induced by an inclined stretching cylinder with non-uniform heat source/sink

**DOI:** 10.1371/journal.pone.0175584

**Published:** 2017-04-25

**Authors:** Tasawar Hayat, Sajid Qayyum, Ahmed Alsaedi, Saleem Asghar

**Affiliations:** 1 Department of Mathematics, Quaid-I-Azam University 45320, Islamabad, Pakistan; 2 Nonlinear Analysis and Applied Mathematics (NAAM) Research Group, Faculty of Science, King Abdulaziz University, Jeddah, Saudi Arabia; 3 Department of Mathematical Sciences, COMSATS Institute of Information Technology, Islamabad, Pakistan; Tianjin University, CHINA

## Abstract

This study investigates the mixed convection flow of Jeffrey liquid by an impermeable inclined stretching cylinder. Thermal radiation and non-uniform heat source/sink are considered. The convective boundary conditions at surface are imposed. Nonlinear expressions of momentum, energy and concentration are transformed into dimensionless systems. Convergent homotopic solutions of the governing systems are worked out by employing homotopic procedure. Impact of physical variables on the velocity, temperature and concentration distributions are sketched and discussed. Numerical computations for skin friction coefficient, local Nusselt and Sherwood numbers are carried out. It is concluded that velocity field enhances for Deborah number while reverse situation is observed regarding ratio of relaxation to retardation times. Temperature and heat transfer rate are enhanced via larger thermal Biot number. Effect of Schmidt number on the concentration and local Sherwood number is quite reverse.

## 1 Introduction

Flow analyses of non-Newtonian liquids have significantly attracted the attention of researchers and scientists during the past few decades [1–5]. It is due to their several applications in geophysics, colloidal and suspension solutions, oil reservoir engineering, bioengineering, chemical and nuclear industries, exotic lubricants, polymer solution, pharmaceuticals, cosmetic processes, paints, paper production etc. Clearly all non-Newtonian materials depend on their effects in shear which cannot be predicted by a single constitutive relationship. This fact of non-Newtonian materials is different than the viscous fluids. Subsequently numerous non-Newtonian liquid models have been recommended for the discussion in view of their diverse characteristics. In general the non-Newtonian materials have been classified into differential, integral and rate types. Available information witnesses that much consideration in the past has been given to the flows of subclasses of differential type materials. It is due to the reason that in differential type materials the shear and normal stresses can be expressed explicitly in terms of velocity components. However this is not true for two and three dimensional flows involving subclasses of rate type fluids. Here we aim to employ the Jeffrey fluid (a subsection of rate type liquids) for the modeling. This model can deliberate for effects of relaxation and retardation times. Few studies dealing with flows of Jeffrey liquid can be mentioned by the refs. [6–10].

The features of stretched surface within frame of mixed convection flow have abundant applications in engineering and industry. Free convection has significance within the light of gravitational force. Heat and mass transfer in flow is also affected by both buoyancy forces and stretching. Such phenomena is important in solar energy systems, cooling of electronic devices, heat exchangers set in a low velocity situation, boilers, atomic reactors cooling during emergency shutdown, cooling of combustion chamber wall in a gas turbine, defroster system, automobile demister and flows in the ocean and atmosphere. Ashraf et al. [11] examined the radiative three-dimensional mixed convection flow of Maxwell fluid by an inclined stretching surface. Mixed convection flow of micropolar liquid in a double stratified medium and chemical reaction is explored by Rashad et al. [12]. Singh and Makinde [13] explored the slip effects in mixed convection flow of viscous fluid towards a moving surface with free stream. Magnetohydrodynamic (MHD) mixed convection flow of viscoelastic fluid past a porous stretching surface was scrutinized by Turkyilmazoglu [14]. Hayat et al. [15] studied mixed convection flow of Jeffrey liquid towards an inclined stretching cylinder with double stratification effects.

Heat transfer radiation is very essential in the design of dependable equipment, gas turbines and several propulsion gadgets for aircraft missiles, nuclear plants, space vehicles and satellites. Likewise the impacts of thermal radiation on the free and forced/constrained convection flows are significant in space technology and procedures including high temperature [16]. Analysis of heat transport radiation of a nanofluid towards stretched surface with temperature jump and velocity slip is addressed by Zheng et al. [17]. Sheikholeslami et al. [18] examined the magnetohydrodynamics (MHD) and radiation phenomena in flow of nanofluid. Hayat et al. [19] studied thermal radiation and heat generation/absorption effects on stagnation point flow of tangent hyperbolic fluid in presence of convective heat and mass transfer. Hayat et al. [20] scrutinized the characteristics of nonlinear thermal radiation and heat source/sink in magnetohydrodynamic (MHD) flow of nanofluid. Some interesting studies for two-phase flows and time series analyses have been presented by Gao et al. [21–25].

Effects of heat source/sink have importance in issues managing with chemical reactions geonuclear repositions and those stressed with dissociating liquids. Heat source/sink can be presumed constant, temperature-dependent or space-dependent. Unsteady magnetohydrodynamic (MHD) flow of pseudoplastic nanofluid in a finite thin film towards a stretching surface with internal heat generation is explored analytically by Lin et al. [26]. Ramesh et al. [27] scrutinized the magnetohydrodynamic (MHD) stagnation point flow of dusty liquid towards a porous stretched surface with non-uniform heat source/sink. Analysis of magnetohydrodynamic (MHD) nonlinear radiative flow of Oldroyd-B nanoliquid in presence of heat generation/absorption is scrutinized by Hayat et al. [28]. Mixed convection flow of Maxwell nanofluid by stretching cylinder with heat source/sink and double stratification is developed by Abbasi et al. [29]. Further in various engineering and industrial procedures the convective surface conditions are more useful in transpiration cooling procedure, material drying etc. The concept of convective surface conditions is initiated by Aziz [30]. He examined the stretched flow of viscous liquid over a convectively heated boundary condition. Characteristics of nonlinear thermal radiation in stagnation point flow of tangent hyperbolic nanofluid with convective convections and chemical reaction are examined by Hayat et al. [31]. Shehzad et al. [32] scrutinized the magnetohydrodynamic (MHD) flow of nanofluid in presence of convective heat and mass conditions. Effect of convective condition in flow of Sisko fluid is developed by Malik et al. [33]. Hayat et al. [34] scrutinized the behavior of thermophoresis and Joule heating in stretched flow of Maxwell liquid over a convectively heated surface.

The purpose of present article is three fold. Firstly to examine non-uniform heat source and sink effects in mixed convection flow of Jeffrey fluid. Secondly to investigate convectively heated cylinder. For this purpose the convective conditions of both heat and mass transfer are considered. Thirdly to address thermal radiation. Therefore our main purpose is to investigate such salient features in the mixed convection flow of Jeffrey liquid by an inclined stretching cylinder. This article is prepared as follows. Mathematical modeling is given in section two. Sections three and four deal with the development and convergence of series solutions. Discussion is arranged in section five. The velocity, temperature and concentration are calculated through homotopy analysis approach [35–40]. Solution expressions for different quantities are analyzed via graphs and tabular values.

## 2 Modeling

**Fig 1:**
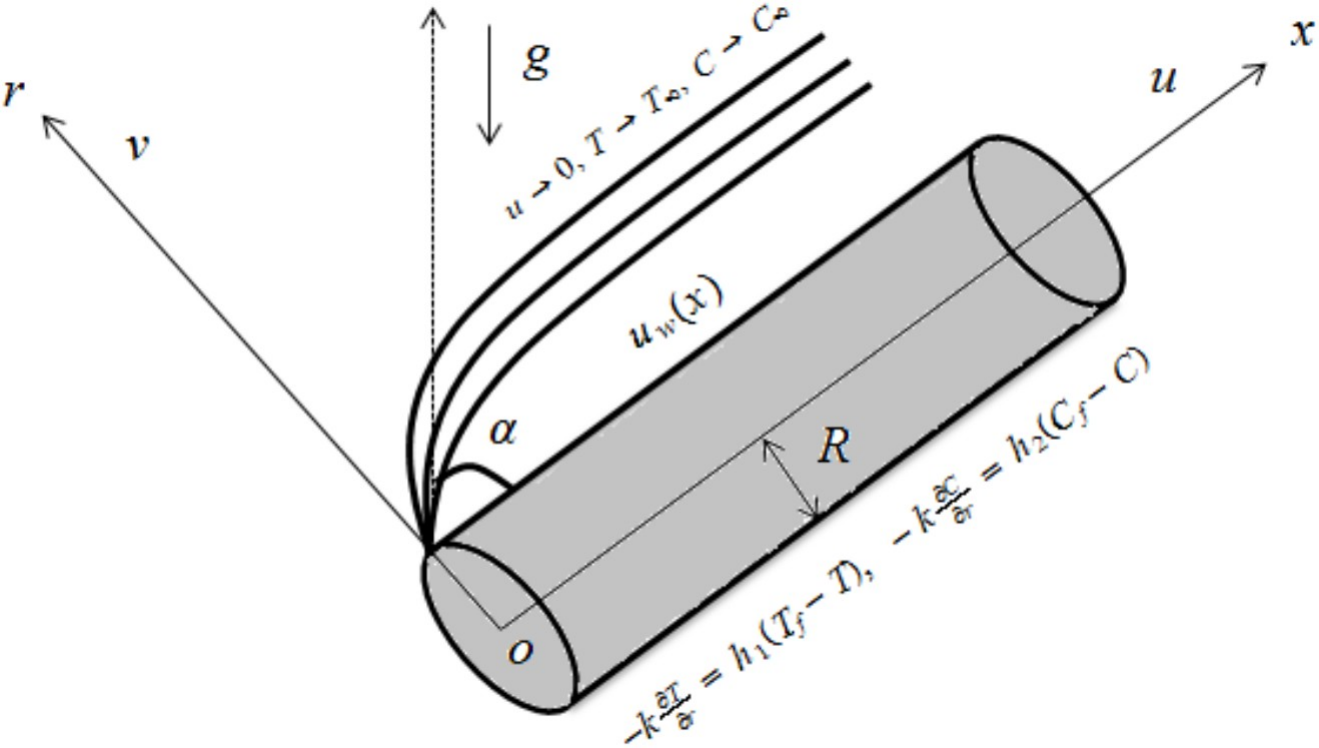
Physical diagram.

We analyze the steady and axisymmetric mixed convection radiative flow of an incompressible Jeffrey fluid due to an inclined cylinder. The physical sketch for the flow is shown in Fig 1. Analysis of heat transfer is deliberated for non-uniform heat source/sink and thermal radiation. Stretching cylinder is taken. Here cylinderical coordinates are used in which *x*–axis is along the axial direction of cylinder and *r*–axis is normal to the cylinder. The convective boundary conditions for a cylinder are employed. The conservation laws after using the boundary layer approximations are reduced into the forms:

$$\frac{\partial (ru)}{\partial x}+\frac{\partial (rv)}{\partial r}=0,$$ (1)

$$\begin{array}{l} u\frac{\partial u}{\partial x}+v\frac{\partial u}{\partial r}=\frac{\nu }{\left(1+\lambda_{1}\right)}\left(\frac{\partial^{2}u}{\partial r^{2}}+\frac{1}{r}\frac{\partial u}{\partial r}\right)+\frac{\nu \lambda_{2}}{\left(1+\lambda_{1}\right)}[v\frac{\partial^{3}u}{\partial r^{3}}+\frac{\partial v}{\partial r}\frac{\partial^{2}u}{\partial r^{2}}\\ \;+u\frac{\partial^{3}u}{\partial x\partial r^{2}}+\frac{\partial u}{\partial r}\frac{\partial^{2}u}{\partial x\partial r}+\frac{1}{r}\left(v\frac{\partial^{2}u}{\partial r^{2}}+u\frac{\partial^{2}u}{\partial x\partial r}\right)]\\ \;+(g\beta_{T}\left(T-T_{\infty }\right)+g\beta_{C}(C-C_{\infty }))\cos\alpha , \end{array}$$ (2)

$$\rho c_{p}\left(u\frac{\partial T}{\partial x}+v\frac{\partial T}{\partial r}\right)=\frac{k}{r}\frac{\partial }{\partial r}\left(r\frac{\partial T}{\partial r}\right)-\frac{1}{r}\frac{\partial }{\partial r}(rq_{r})+q^{\prime \prime \prime },$$ (3)

$$u\frac{\partial C}{\partial x}+v\frac{\partial C}{\partial r}=D(\frac{\partial^{2}C}{\partial r^{2}}+\frac{1}{r}\frac{\partial C}{\partial r}).$$ (4)

The associated boundary conditions are

$$\begin{array}{l} u(x,r)=u_{w}\left(x\right)=\frac{u_{o}x}{l},\;v(x,r)=0,\;-k\frac{\partial T}{\partial r}=h_{t}(T_{f}-T),\\ -D\frac{\partial C}{\partial r}=h_{c}(C_{f}-C)\;\text{at}\;r=R,\\ u(x,r)\to 0,\;T\to T_{\infty },\;C\to C_{\infty }\;\text{as}\;r\to \infty . \end{array}$$ (5)

Here we see that second and third terms on the right hand side (R. H. S) in Eq (3) show the thermal radiation and non-uniform heat source/sink. We denote (*u*, *v*) the components of velocity in the (*x*, *r*) directions, *ν* = (*μ*/*ρ*) the fluid kinematic viscosity, *μ* the dynamic viscosity of fluid, *λ*_1_ the relaxation to retardation times ratio, *g* the gravitational acceleration, *λ*_2_ the retardation time, *ρ* the fluid density, *α* the angle of inclination, *β*_*T*_ the coefficient of thermal expansion and *β*_*C*_ the coefficient of concentration expansion, *c*_*p*_ the specific heat, *k* the thermal conductivity, *q*‴ the non-uniform heat source/sink, *u*_*w*_(*x*) the linear stretching velocity, *u*_0_ the reference velocity, *q*_*r*_ the radiative heat flux, *T*_*f*_ the convective fluid temperature, *C*_*f*_ the convective fluid concentration, *h*_*t*_ the coefficient of convective heat transfer, *h*_*c*_ the coefficient of convective mass transfer, *T* and *T*_∞_ the fluid and ambient temperatures, *D* the mass diffusivity, *C* and *C*_∞_ the fluid and ambient concentrations respectively and *l* the characteristic length.

Radiative heat flux *q*_*r*_ through Rosseland approximation is given as follows [5]:

$$q_{r}=-\frac{4\sigma^{*}}{3k^{*}}\frac{\partial T^{4}}{\partial r},$$ (6)

where σ^*^ denotes the Stefan-Boltzmann constant and k^*^ shows mean absorption coefficient. We assume that the difference of temperature inside the flow is such that the term *T*^4^ might be characterized as a linear function of temperature. This is achieved by expanding *T*^4^ in a Taylor series about *T*_∞_ as follows:

$$T^{4}=T_{\infty }^{4}+4T_{\infty }^{3}(T-T_{\infty })+6T_{\infty }^{2}{\left(T-T_{\infty }\right)}^{2}+\ldots \ldots \ldots$$ (7)

Neglecting higher order terms we obtain

$$T^{4}≅4T_{\infty }^{3}T-3T_{\infty }^{4}.$$ (8)

From Eqs (8) and (6) one has

$$q_{r}=-\frac{16\sigma^{*}T_{\infty }^{3}}{3k^{*}}\frac{\partial T}{\partial r}.$$ (9)

Further Eqs (3) and (9) yield

$$\rho c_{p}\left(u\frac{\partial T}{\partial x}+v\frac{\partial T}{\partial r}\right)=\frac{k}{r}\frac{\partial }{\partial r}\left(r\frac{\partial T}{\partial r}\right)+\frac{16\sigma^{*}T_{\infty }^{3}}{3k^{*}}\left(\frac{1}{r}\frac{\partial T}{\partial r}+\frac{\partial^{2}T}{\partial r^{2}}\right)+q^{\prime \prime \prime }.$$ (10)

Non-uniform heat source/sink *q*‴ satisfies [27]:

$$q^{\prime \prime \prime }=\frac{ku_{w}\left(x\right)}{x\nu }\left[A(T_{f}-T_{\infty })f^{\prime }+(T-T_{\infty })B\right],$$ (11)

where *A* is taken regarding space-dependent coefficient and *B* for temperature-dependent coefficient of the heat source/sink. Note that the case (*A* > 0, *B* > 0) relates to internal heat generation and (*A* < 0, *B* < 0) for internal heat absorption.

The appropriate transformations are

$$\begin{array}{l} \eta =\sqrt{\frac{u_{0}}{\nu l}}\left(\frac{r^{2}-R^{2}}{2R}\right),\;\theta \left(\eta \right)=\frac{T-T_{\infty }}{T_{f}-T_{\infty }},\;\phi \left(\eta \right)=\frac{C-C_{\infty }}{C_{f}-C_{\infty }},\\ u=\frac{u_{0}x}{l}f^{\prime }\left(\eta \right),\;v=-\frac{R}{r}\sqrt{\frac{u_{0}\nu }{l}}f\left(\eta \right),\;\psi (\eta )=\sqrt{\frac{u_{0}\nu x^{2}}{l}}Rf(\eta ). \end{array}$$ (12)

Using Eq (12), Eq (1) is trivially satisfied and Eqs (2), (4), (5) and (10) become

$$\begin{array}{c} \left(1+2\gamma \eta \right)f^{\prime \prime \prime }+2\gamma f^{\prime \prime }+(1+\lambda_{1})(ff^{\prime \prime }-{\left(f^{\prime }\right)}^{2})+\gamma \beta \left(f^{\prime }f^{\prime \prime }-3ff^{\prime \prime \prime }\right)\\ +\beta \left(1+2\gamma \eta \right)\left({\left(f^{\prime \prime }\right)}^{2}-ff^{iv}\right)+(1+\lambda_{1})\lambda_{T}(\theta +N\phi )\cos\alpha =0, \end{array}$$ (13)

$$\left(1+\frac{4}{3}R\right)\left(\left(1+2\gamma \eta \right)\theta^{\prime \prime }+2\gamma \theta^{\prime }\right)+\mathrm{Pr}f\theta^{\prime }+Af^{\prime }+B\theta =0,$$ (14)

$$\left(1+2\gamma \eta \right)\phi^{\prime \prime }+2\gamma \phi^{\prime }+Scf\phi^{\prime }=0,$$ (15)

$$f(\eta )=0,\;f^{\prime }(\eta )=1,\;\theta^{\prime }(0)=-Bi_{1}(1-\theta (0)),\;\phi^{\prime }(0)=-Bi_{2}(1-\phi (0))\;\text{at}\;\eta =0,$$

$$f^{\prime }(\eta )=0,\;\theta (\eta )=0,\;\phi (\eta )=0\;\text{as}\;\eta \to \infty .$$ (16)

Here prime shows differentiation with respect to *η*, *γ* for curvature parameter, *λ*_1_ for relaxation to retardation times ratio, *β* for Deborah number (in view of retardation time), *λ*_*T*_ for mixed convection (or thermal buoyancy) parameter, *N* for ratio of concentration to thermal buoyancy forces, *α* for angle of inclination, *R* for radiation parameter, Pr for Prandtl number, *Sc* for Schmidt number, *Bi*_1_ and *Bi*_2_ for Biot numbers due to heat and mass transfer, *Gr* for temperature Grashof number and Gr^*^ for mass Grashof number. These parameters are defined by

$$\begin{array}{l} \;\gamma =\sqrt{\frac{\nu l}{u_{0}R^{2}}},\;\beta =\frac{\lambda_{2}u_{0}}{l},\;\lambda_{T}=\frac{Gr}{Re_{x}^{2}},\;N=\frac{Gr^{*}}{Gr}=\frac{\beta_{C}(C_{f}-C_{\infty })}{\beta_{T}\left(T_{f}-T_{\infty }\right)},\\ \;R=\frac{4\sigma^{*}T_{\infty }^{3}}{kk^{*}},\;\mathrm{Pr}=\frac{\mu c_{p}}{k},\;Sc=\frac{\nu }{D},\;Bi_{1}=\frac{h_{t}}{k}\sqrt{\frac{u_{0}}{\nu l}},\;Bi_{2}=\frac{h_{c}}{D}\sqrt{\frac{u_{0}}{\nu l}},\;\\ Gr=\frac{g\beta_{T}\left(T_{f}-T_{\infty }\right)x^{3}}{\nu^{2}},\;Gr^{*}=\frac{g\beta_{C}(C_{f}-C_{\infty })x^{3}}{\nu^{2}}. \end{array}$$ (17)

Note that the conditions in Eq (16) are reduced to heat and mass flux conditions when *Bi*_1_ → ∞ and *Bi*_2_ → ∞. The skin friction coefficient *C*_*f*_, local Nusselt *Nu*_*x*_ and Sherwood *Sh*_*x*_ numbers are defined in dimensional forms as follows:

$$C_{f}=\frac{\tau_{w}}{\frac{1}{2}\rho u_{w}^{2}},\;Nu_{x}=\frac{xq_{w}}{k(T_{f}-T_{\infty })},\;Sh_{x}=\frac{xj_{w}}{D(C_{f}-C_{\infty })},$$ (18)

in which *τ*_*w*_ (surface shear stress), *q*_*w*_ (surface heat flux) and *j*_*w*_ (surface mass flux) are given by

$$\begin{array}{l} \tau_{w}=\frac{\mu }{\left(1+\lambda_{1}\right)}{\left(\frac{\partial u}{\partial r}+\lambda_{2}\left(v\frac{\partial^{2}u}{\partial r^{2}}+u\frac{\partial^{2}u}{\partial x\partial r}\right)\right)}_{r=R},\\ q_{w}=-\left(k+\frac{16\sigma^{*}T_{\infty }^{3}}{3k^{*}}\right){\left(\frac{\partial T}{\partial r}\right)}_{r=R},\;j_{w}=-D{\left(\frac{\partial C}{\partial r}\right)}_{r=R}. \end{array}$$ (19)

Skin friction coefficient *C*_*f*_, local Nusselt number *Nu*_*x*_ and local Sherwood number *Sh*_*x*_ are

$$\begin{array}{l} \frac{1}{2}Re_{x}^{0.5}C_{f_{x}}=\frac{1}{\left(1+\lambda_{1}\right)}(f^{\prime \prime }(0)+\beta (-f(0)f^{\prime \prime \prime }(0)-\gamma f(0)f^{\prime \prime }(0)+f^{\prime }(0)f^{\prime \prime }(0))),\\ Re_{x}^{-0.5}Nu_{x}=-\left(1+\frac{4}{3}R\right)\theta^{\prime }\left(0\right),\;Re_{x}^{-0.5}Sh_{x}=-\phi^{\prime }(0), \end{array}$$ (20)

in which $Re_{x}=\frac{u_{o}x^{2}}{\nu l}$ is the local Reynolds number.

## 3 Homotopy analysis procedure

Here we take initial approximations (*f*_0_, *θ*_0_, *ϕ*_0_) and auxiliary linear operators (**L**_*f*_, **L**_*θ*_, **L**_*ϕ*_) for the momentum, energy and concentration equations in the forms

$$f_{0}\left(\eta \right)=1-\exp\left(-\eta \right),\;\theta_{0}\left(\eta \right)=\frac{Bi_{1}}{1+Bi_{1}}\exp\left(-\eta \right),\;\phi_{0}(\eta )=\frac{Bi_{2}}{1+Bi_{2}}\exp(-\eta ),$$ (21)

$$L_{f}\left(f\right)=\frac{d^{3}f}{d\eta^{3}}-\frac{df}{d\eta },\;L_{\theta }\left(\theta \right)=\frac{d^{2}\theta }{d\eta^{2}}-\theta ,\;L_{\phi }\left(\phi \right)=\frac{d^{2}\phi }{d\eta^{2}}-\phi ,$$ (22)

with the associated properties

$$L_{f}\left[A_{1}+A_{2}\exp(-\eta )+A_{3}\exp(\eta )\right]=0,$$ (23)

$$L_{\theta }\left[A_{4}\exp(-\eta )+A_{5}\exp(\eta )\right]=0,$$ (24)

$$L_{\phi }\left[A_{6}\exp(-\eta )+A_{7}\exp(\eta )\right]=0,$$ (25)

in which *A*_*i*_ (*i* = 1–7) are the arbitrary constants. The zeroth-order and *m*th-order deformation systems are constructed as follows:

### 3.1 Zeroth-order systems

The relevant problems are

$$\left(1-p\right)L_{f}\left[\overset{⌢}{f}\left(\eta ;p\right)-f_{0}\left(\eta \right)\right]=pH_{f}\hbar_{f}N_{f}\left[\overset{⌢}{f}\left(\eta ;p\right),\;\overset{⌢}{\theta }\left(\eta ;p\right),\;\overset{⌢}{\phi }\left(\eta ;p\right)\right],$$ (26)

$$\left(1-p\right)L_{\theta }\left[\overset{⌢}{\theta }\left(\eta ;p\right)-\theta_{0}\left(\eta \right)\right]=pH_{\theta }\hbar_{\theta }N_{\theta }\left[\overset{⌢}{\theta }\left(\eta ;p\right),\overset{⌢}{f}\left(\eta ;p\right)\right],$$ (27)

$$\left(1-p\right)L_{\phi }\left[\overset{⌢}{\phi }\left(\eta ;p\right)-\phi_{0}\left(\eta \right)\right]=pH_{\phi }\hbar_{\phi }N_{\phi }\left[\overset{⌢}{\phi }\left(\eta ;p\right),\overset{⌢}{f}\left(\eta ;p\right)\right],$$ (28)

f⌢(0;p)=0,f⌢'(0;p)=1andf⌢'(η;p)→0asη→∞, (29)

θ⌢'(0;p)=−Bi1(1−θ⌢(0;p))andθ⌢(η;p)→0asη→∞, (30)

ϕ⌢'(0;p)=−Bi2(1−ϕ⌢(0;p))andϕ⌢(η;p)→0asη→∞, (31)

Nf[f⌢(η;p),θ⌢(η;p),ϕ⌢(η;p)]=(1+2γη)f⌢'''+2γf⌢''+(1+λ1)(f⌢f⌢''−(f⌢')2)+γβ(f⌢'f⌢''−3f⌢f⌢''')+β(1+2γη)((f⌢'')2−f⌢f⌢iv)+(1+λ1)λT(θ⌢+Nϕ⌢)cosα, (32)

Nθ[θ⌢(η;p),f⌢(η;p)]=(1+43R)((1+2γη)θ⌢''+2γθ⌢')+Prf⌢θ⌢'+Af⌢'+Bθ⌢, (33)

Nϕ[ϕ⌢(η;p),f⌢(η;p)]=(1+2γη)ϕ⌢''+2γϕ⌢'+Scf⌢ϕ⌢'. (34)

Here *p* ∈ [0,1] is embedding parameter, *H*_*f*_, *H*_*θ*_, *H*_*ϕ*_ the auxiliary functions and ℏ_*f*_, ℏ_*θ*_, ℏ_*ϕ*_ the non-zero auxiliary variables.

### 3.2 *m*th-order deformation systems

Here we have

$$L_{f}\left[f_{m}\left(\eta \right)-\chi_{m}f_{m-1}\left(\eta \right)\right]=\hbar_{f}R_{m}^{f}\left(\eta \right),$$ (35)

$$L_{\theta }\left[\theta_{m}\left(\eta \right)-\chi_{m}\theta_{m-1}\left(\eta \right)\right]=\hbar_{\theta }R_{m}^{\theta }\left(\eta \right),$$ (36)

$$L_{\phi }\left[\phi_{m}\left(\eta \right)-\chi_{m}\phi_{m-1}\left(\eta \right)\right]=\hbar_{\phi }R_{m}^{\phi }\left(\eta \right),$$ (37)

$$f_{m}\left(0\right)=0,\;f_{m}^{\prime }\left(0\right)=0\;\text{and}\;f_{m}^{\prime }\left(\eta \right)\to 0\;\text{as}\;\eta \to \infty ,$$

$$\theta_{m}^{\prime }(0)-Bi_{1}\theta_{m}(0)=0\;\text{and}\;\theta_{m}\left(\eta \right)\to 0\;\text{as}\;\eta \to \infty ,$$ (38)

$$\phi_{m}^{\prime }(0)-Bi_{2}\phi_{m}(0)=0\;\text{and}\;\phi_{m}\left(\eta \right)\to 0\;\text{as}\;\eta \to \infty ,$$

$$\begin{array}{l} R_{m}^{f}\left(\eta \right)=\left(1+2\gamma \eta \right)f_{m-1}^{\prime \prime \prime }\left(\eta \right)+2\gamma f_{m-1}^{\prime \prime }\left(\eta \right)+\sum_{k=0}^{m-1}\left[\left(1+\lambda_{1})(f_{m-1-k}f_{k}^{\prime \prime }-f_{m-1-k}^{\prime }f_{k}^{\prime }\right)\right]\\ \;+\sum_{k=0}^{m-1}\left[\gamma \beta \left(f_{m-1-k}^{\prime }f_{k}^{\prime \prime }-3f_{m-1-k}f_{k}^{\prime \prime \prime }\right)+\beta \left(1+2\gamma \eta \right)\left(f_{m-1-k}^{\prime \prime }f_{k}^{\prime \prime }-f_{m-1-k}f_{k}^{iv}\right)\right]\\ \;+(1+\lambda_{1})\lambda_{T}(\theta_{m-1}+N\phi_{m-1})\cos\alpha , \end{array}$$ (39)

$$R_{m}^{\theta }\left(\eta \right)=\left(1+\frac{4}{3}R\right)\left(\left(1+2\gamma \eta \right)\theta_{m-1}^{\prime \prime }\left(\eta \right)+2\gamma \theta_{m-1}^{\prime }(\eta )\right)+\mathrm{Pr}\overset{m-1}{\underset{k=0}{\sum }}f_{m-1-k}\theta_{k}^{\prime }+Af_{m-1}^{\prime }+B\theta_{m-1},$$ (40)

$$R_{m}^{\phi }\left(\eta \right)=\left(1+2\gamma \eta \right)\phi_{m-1}^{\prime \prime }\left(\eta \right)+2\gamma \phi_{m-1}^{\prime }(\eta )+Sc\overset{m-1}{\underset{k=0}{\sum }}f_{m-1-k}\phi_{k}^{\prime },$$ (41)

$$\chi_{m}=\{\begin{array}{c} 0,\;m\leq 1,\\ 1,\;m>1. \end{array}$$ (42)

For setting *p* = 0 and *p* = 1, we can write

$$\overset{⌢}{f}\left(\eta ;0\right)=f_{0}\left(\eta \right),\;\overset{⌢}{f}\left(\eta ;1\right)=f\left(\eta \right),$$ (43)

$$\overset{⌢}{\theta }\left(\eta ;0\right)=\theta_{0}\left(\eta \right),\;\overset{⌢}{\theta }\left(\eta ;1\right)=\theta \left(\eta \right),$$ (44)

$$\overset{⌢}{\phi }\left(\eta ;0\right)=\phi_{0}\left(\eta \right),\;\overset{⌢}{\phi }\left(\eta ;1\right)=\phi \left(\eta \right).$$ (45)

When *p* increases from 0 to 1 then $\overset{⌢}{f}\left(\eta ;p\right)$, $\overset{⌢}{\theta }\left(\eta ;p\right)$ and $\overset{⌢}{\phi }\left(\eta ;p\right)$ vary from the initial approximations *f*_0_(*η*), *θ*_0_(*η*) and *ϕ*_0_(*η*) to the desired solutions *f*(*η*), *θ*(*η*) and *ϕ*(*η*). Using Taylor expansion and considering the convergence of the series solutions at *p* = 1 we have

$$f\left(\eta \right)=f_{0}\left(\eta \right)+\overset{\infty }{\underset{m=1}{\sum }}f_{m}\left(\eta \right),$$ (46)

$$\theta \left(\eta \right)=\theta_{0}\left(\eta \right)+\overset{\infty }{\underset{m=1}{\sum }}\theta_{m}\left(\eta \right),$$ (47)

$$\phi \left(\eta \right)=\phi_{0}\left(\eta \right)+\overset{\infty }{\underset{m=1}{\sum }}\phi_{m}\left(\eta \right).$$ (48)

The general solutions (*f*_*m*_, *θ*_*m*_, *ϕ*_*m*_) of Eqs (35–37) in view of particular solutions $\left(f_{m}^{*},\;\theta_{m}^{*},\;\phi_{m}^{*}\right)$ are

$$f_{m}\left(\eta \right)=f_{m}^{*}\left(\eta \right)+A_{1}+A_{2}\exp(-\eta )+A_{3}\exp(\eta ),$$ (49)

$$\theta_{m}\left(\eta \right)=\theta_{m}^{*}\left(\eta \right)+A_{4}\exp(-\eta )+A_{5}\exp(\eta ),$$ (50)

$$\phi_{m}\left(\eta \right)=\phi_{m}^{*}\left(\eta \right)+A_{6}\exp(-\eta )+A_{7}\exp(\eta ).$$ (51)

Using the boundary conditions (38), the values of constants *A*_*i*_ (*i* = 1–7) are

$$\begin{array}{l} A_{1}=-{\frac{\partial f_{m}^{*}\left(\eta \right)}{\partial \eta }|}_{\eta =0}-f_{m}^{*}(0),\;A_{2}={\frac{\partial f_{m}^{*}\left(\eta \right)}{\partial \eta }|}_{\eta =0},\;A_{3}=0,\\ A_{4}=\frac{1}{Bi_{1}+1}\left[{\frac{\partial \theta_{m}^{*}\left(\eta \right)}{\partial \eta }|}_{\eta =0}-Bi_{1}\theta_{m}^{*}\left(0\right)\right],\;A_{5}=0,\\ A_{6}=\frac{1}{Bi_{2}+1}\left[{\frac{\partial \phi_{m}^{*}\left(\eta \right)}{\partial \eta }|}_{\eta =0}-Bi_{2}\phi_{m}^{*}\left(0\right)\right],\;A_{7}=0. \end{array}$$ (52)

### 3.3 Convergence of the series solutions

**Fig 2:**
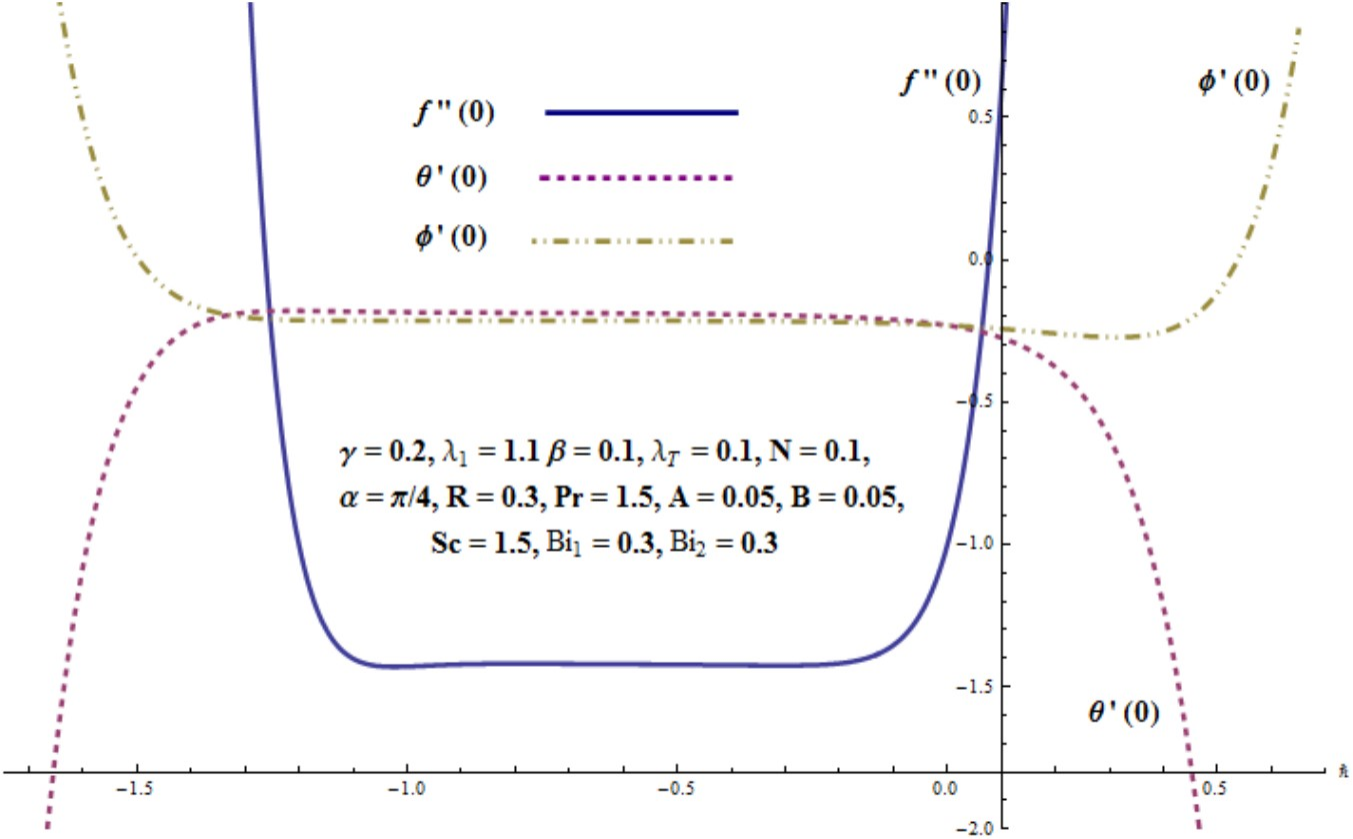
ℏ-curves for *f*"(0), *θ*'(0) and *ϕ*'(0).

Clearly the obtained series solutions consist of auxiliary parameter ℏ. This auxiliary parameter ℏ has vital role to adjust and control the convergence region of the homotopic solutions. Thus the ℏ-curves for admissible values of convergence control parameters are displayed (see Fig 2). It is observed that acceptable ranges of ℏ_*f*_, ℏ_*θ*_ and ℏ_*ϕ*_ are −0.95 ≤ ℏ_*f*_ ≤ −0.3, −1.35 ≤ ℏ_*θ*_ ≤ −0.25 and −1.2 ≤ ℏ_*ϕ*_ ≤ −0.25.

## 3.4 Discussion

**Fig 3:**
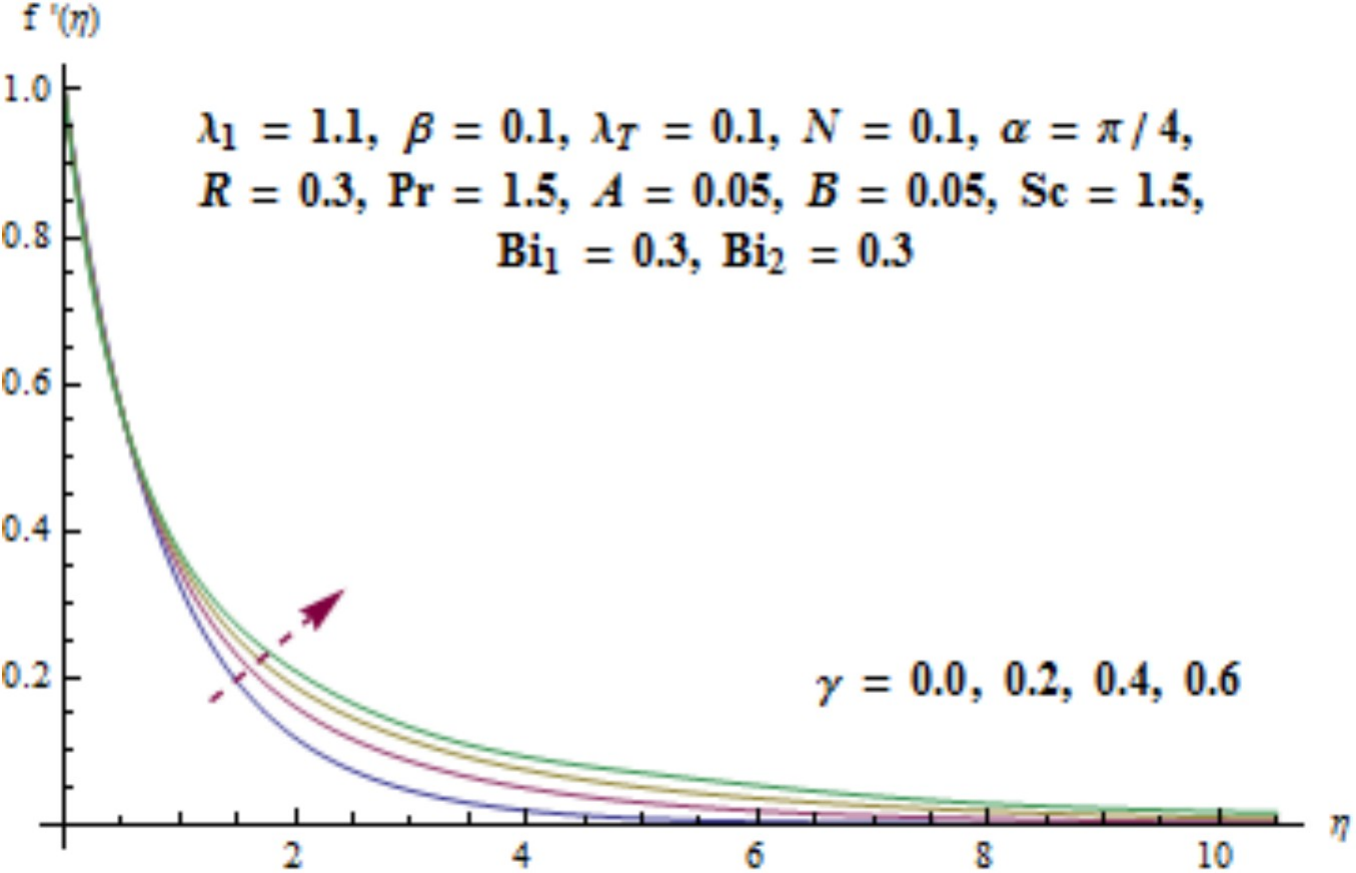
Behavior of *γ* on *f*'(*η*).

**Fig 4:**
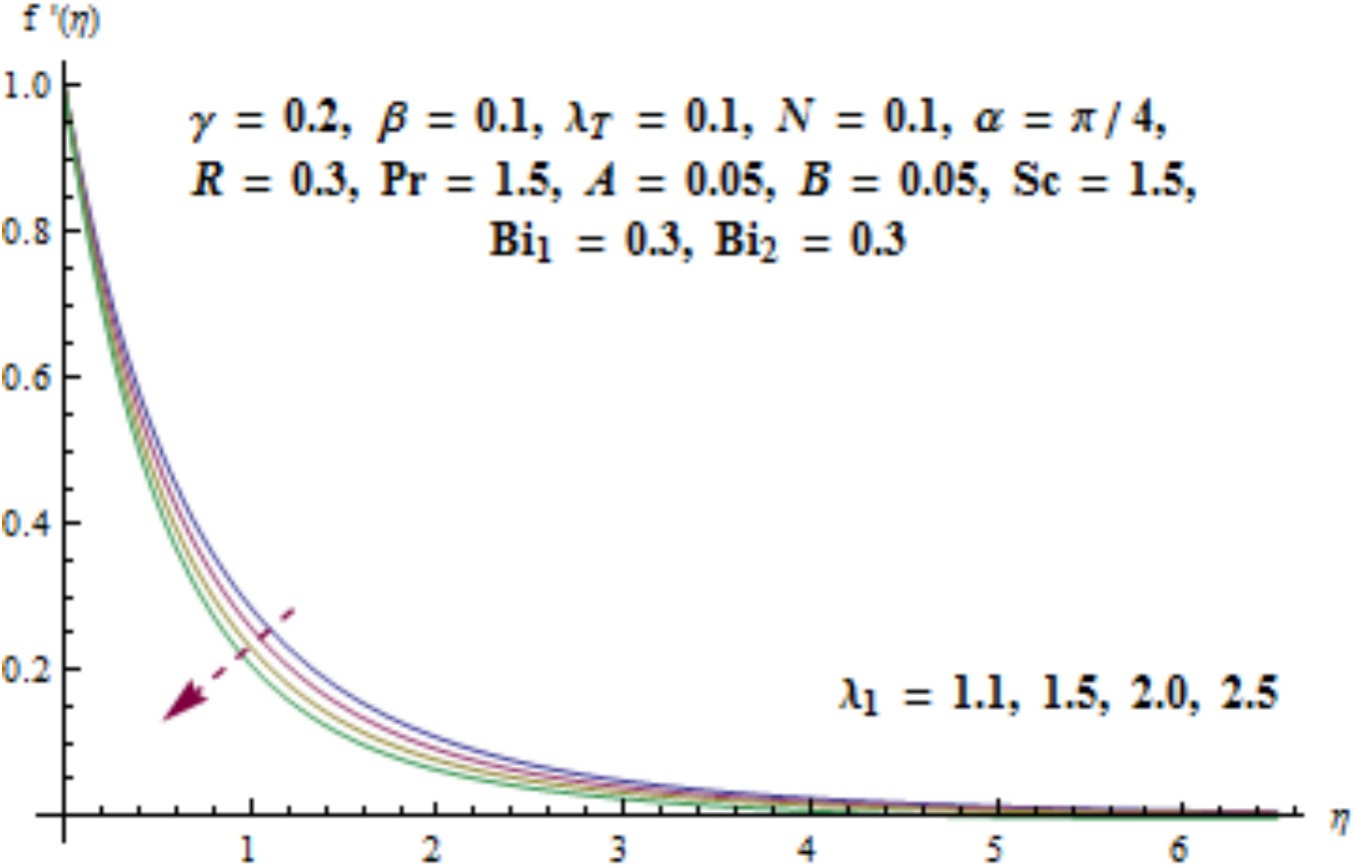
Behavior of *λ*_1_ on *f*'(*η*).

**Fig 5:**
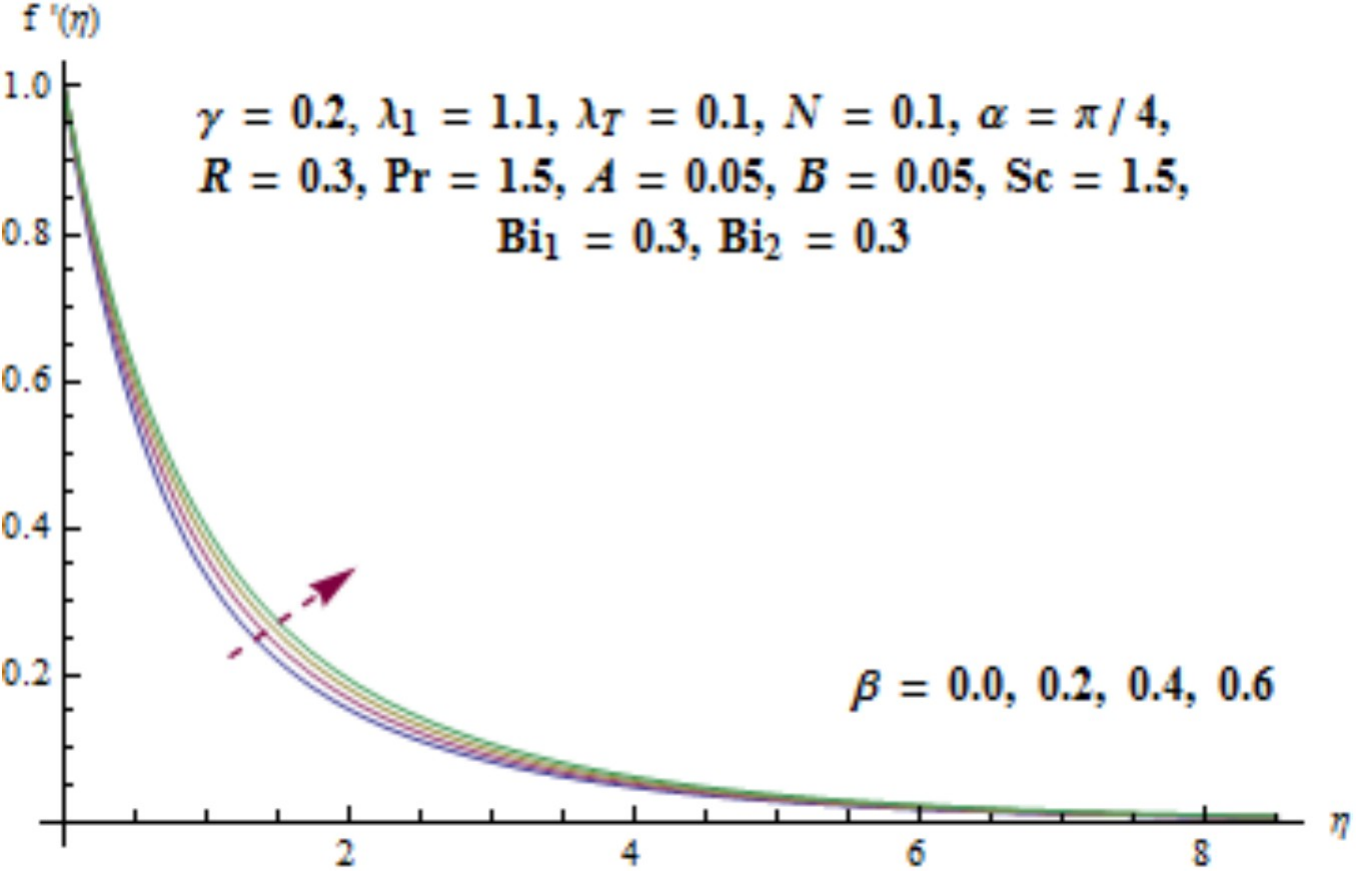
Behavior of *β* on *f*'(*η*).

**Fig 6:**
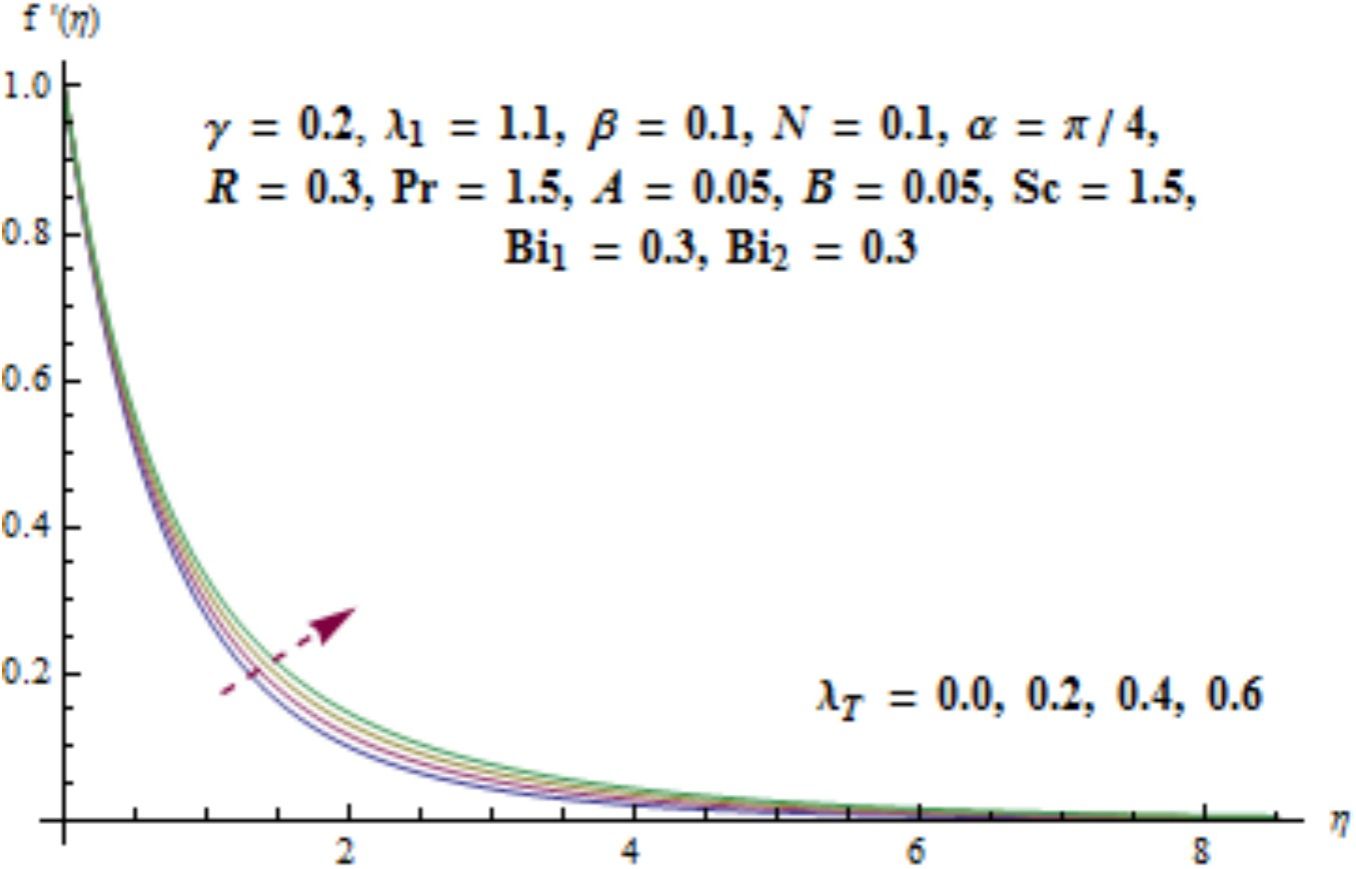
Behavior of *λ*_*T*_ on *f*'(*η*).

**Fig 7:**
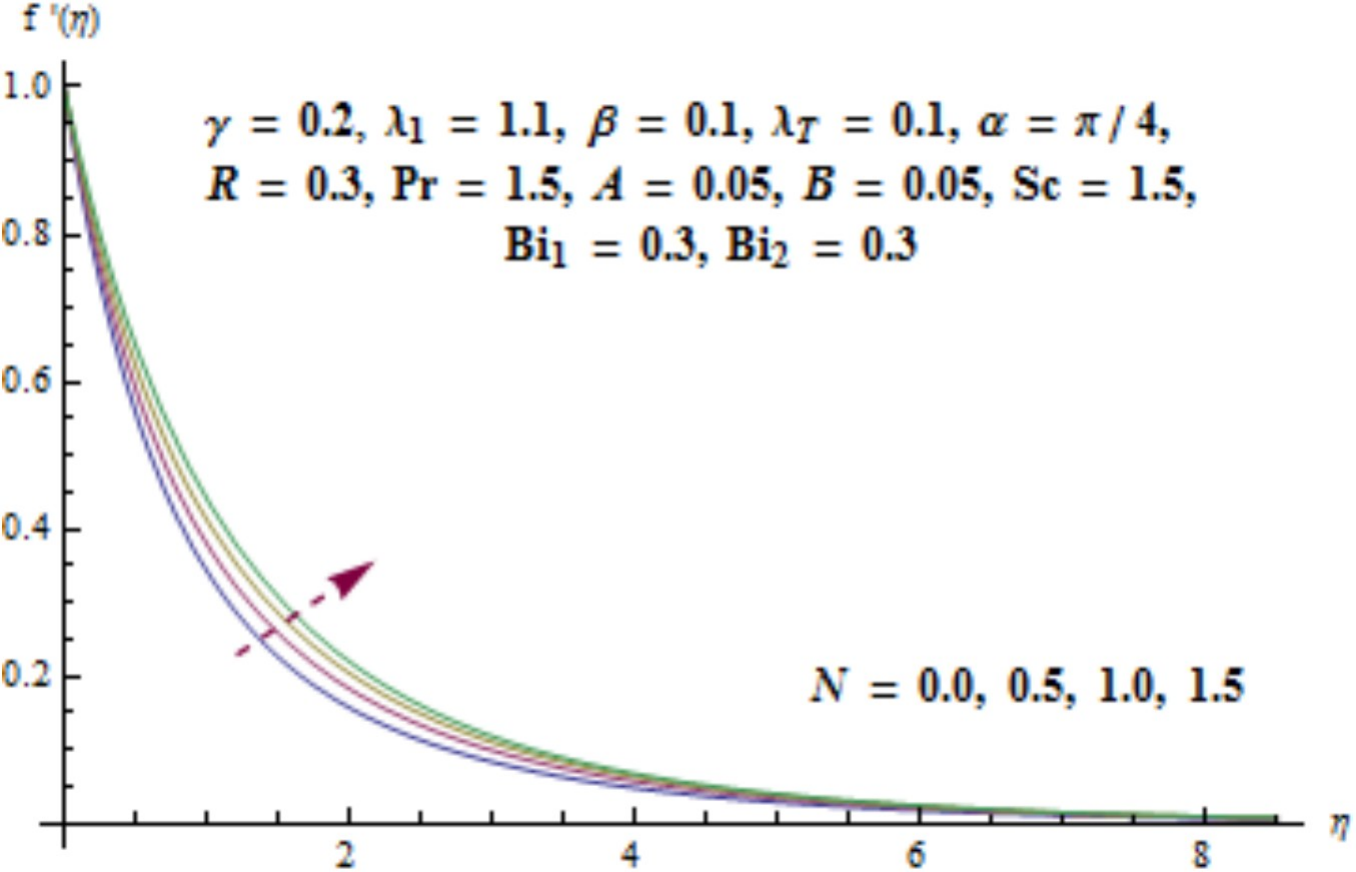
Behavior of *N* on *f*'(*η*).

**Fig 8:**
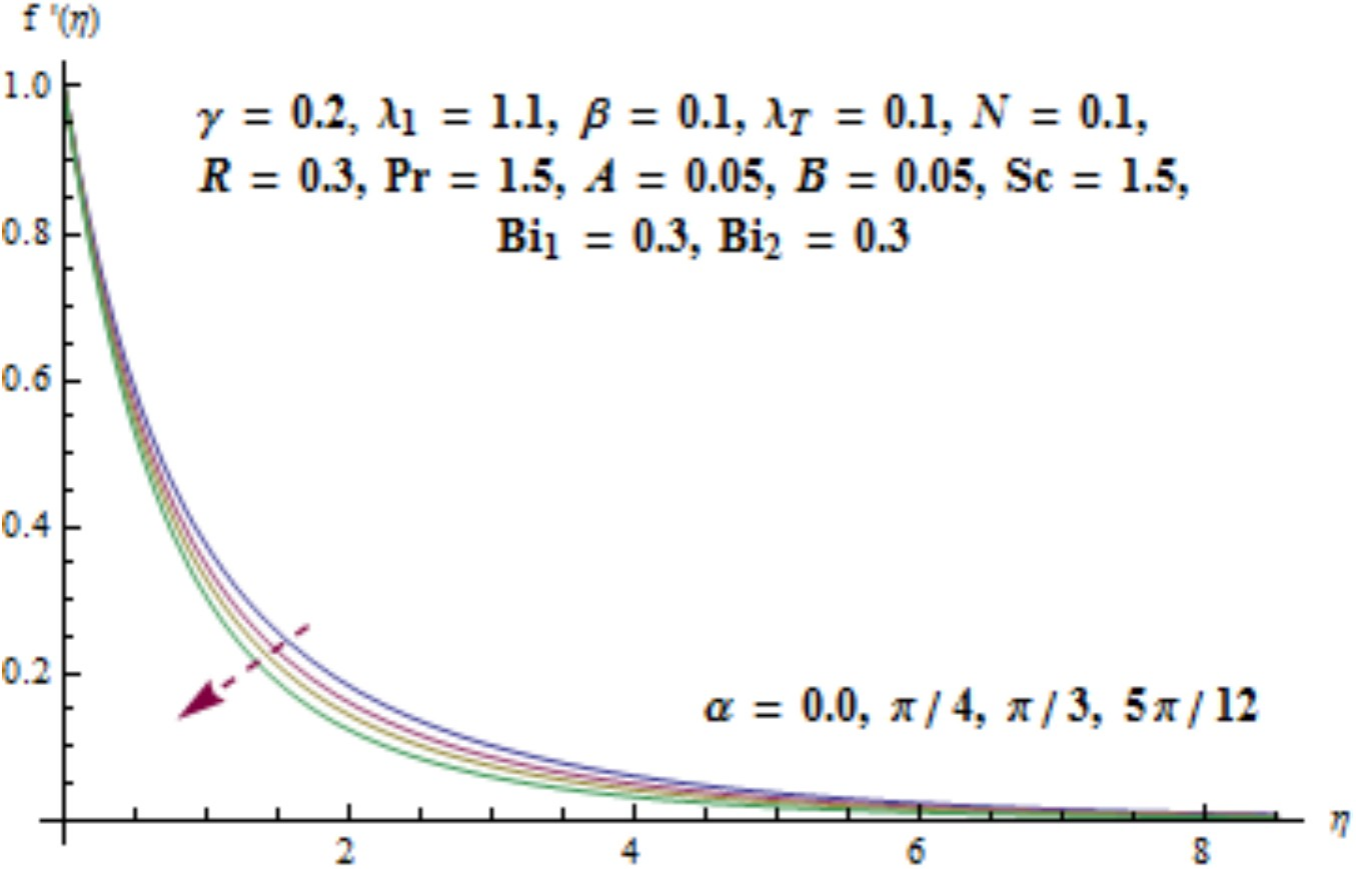
Behavior of *α* on *f*'(*η*).

This section aims to investigate the behavior of various pertinent quantities on the velocity, temperature and concentration fields. Fig 3 is sketched for the behavior of curvature parameter *γ* on velocity distribution. It is found that velocity field has decreasing behavior near the cylinder while it enhances far away from it. It is noted that thickness of boundary layer enhances. Physically it is verified that larger values of curvature parameter *γ* decrease the cylinder radius. Consequently contact surface area of cylinder with the liquid decreases which offers less resistance to the motion of fluid and ultimate velocity enhances. Variation of *λ*_1_ (i.e., the relaxation to retardation times ratio) on velocity is plotted in Fig 4. It is shown that velocity field decays for higher values of *λ*_1_. Since *λ*_1_ is the relaxation and retardation times ratio so for larger values of *λ*_1_ the relaxation time enhances which provides additional resistance to the fluid motion. Hence velocity field decreases. Effect of Deborah number *β* on the velocity distribution is sketched in Fig 5. There is an enhancement in the velocity and thickness of momentum layer when *β* increases. With larger Deborah number *β* the retardation time enhances. It leads to an enhancement in velocity. Variation of mixed convection parameter *λ*_*T*_ on velocity is sketched in Fig 6. Larger mixed convection parameter *λ*_*T*_ correspond to an increase in velocity and thickness of momentum layer. Physically we observed that an increase in mixed convection parameter *λ*_*T*_ corresponds to an enhancement of thermal buoyancy force which is responsible for higher velocity. Characteristics of *N* i.e., concentration to thermal buoyancy forces ratio on the velocity are plotted in Fig 7. It is analyzed that velocity field and associated boundary layer thickness are enhanced for higher values of *N*. Since *N* is the concentration and thermal buoyancy forces ratio so with the increment of *N* the concentration buoyancy force enhances which results in upgradation of velocity. Fig 8 elucidates the effect of angle of inclination *α* on velocity field. Larger values of *α* results in the reduction of velocity field. In fact through higher *α* the gravity affect decreases which results in the decrease of velocity profile.

**Fig 9:**
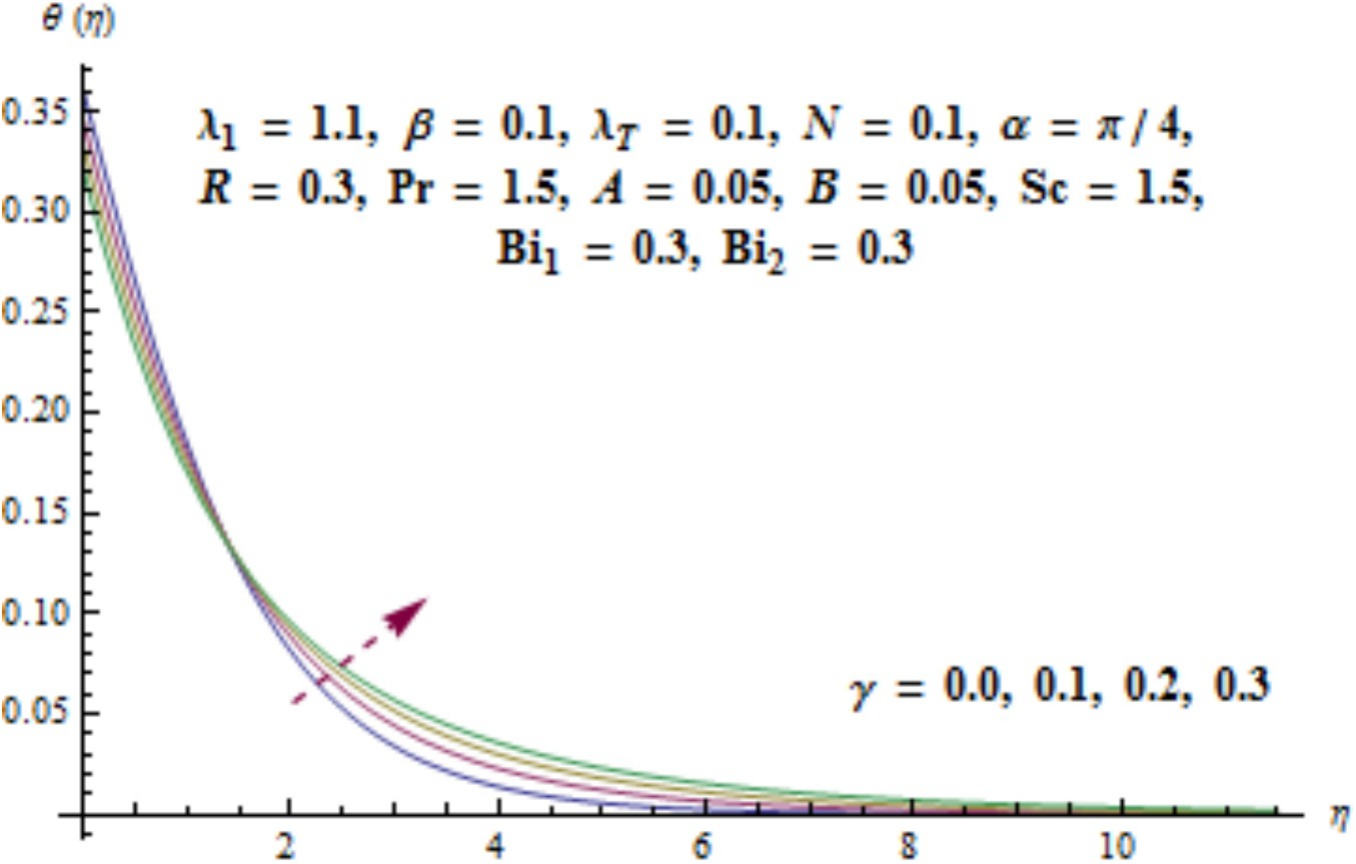
Behavior of *γ* on *θ*(*η*).

**Fig 10:**
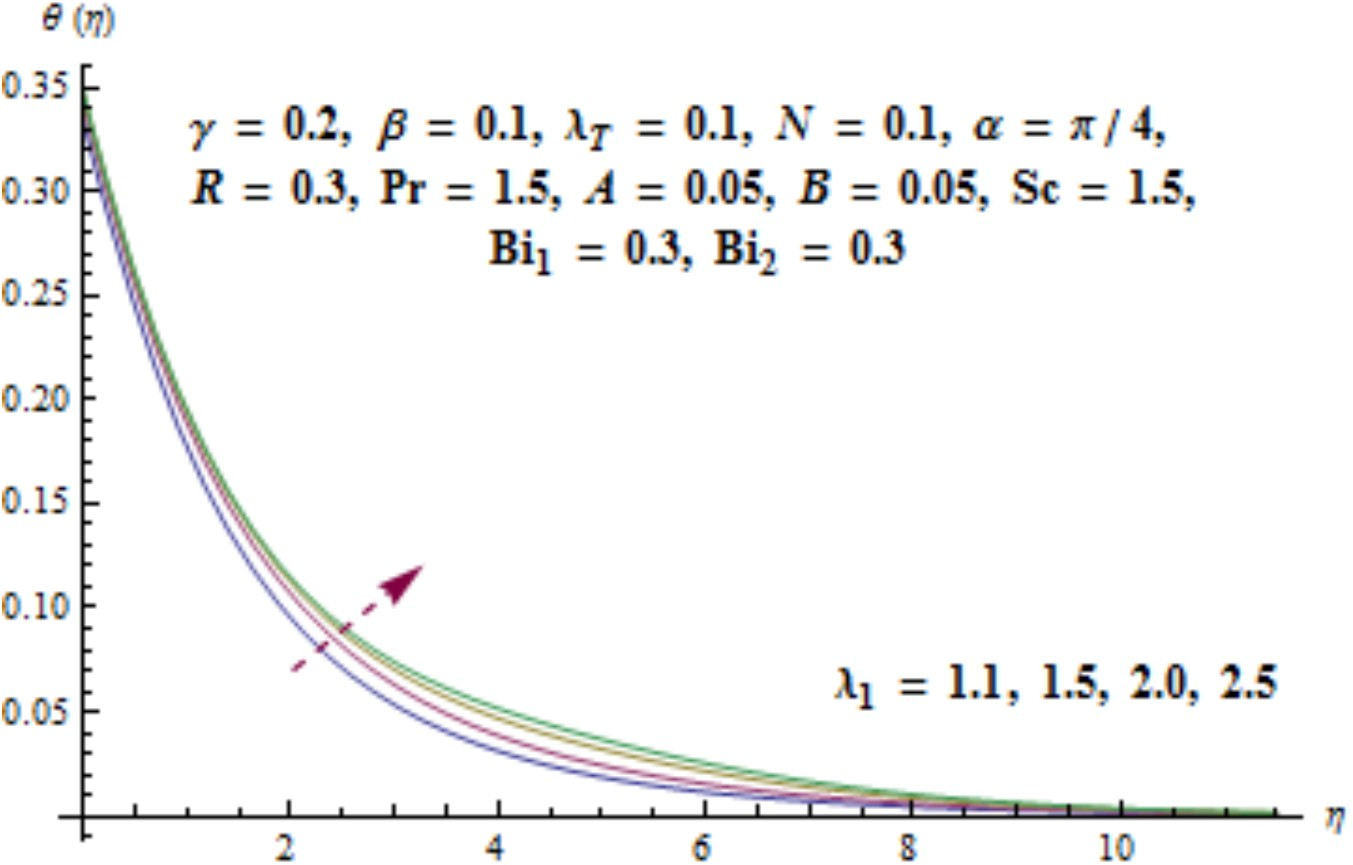
Behavior of *λ*_1_ on *θ*(*η*).

**Fig 11:**
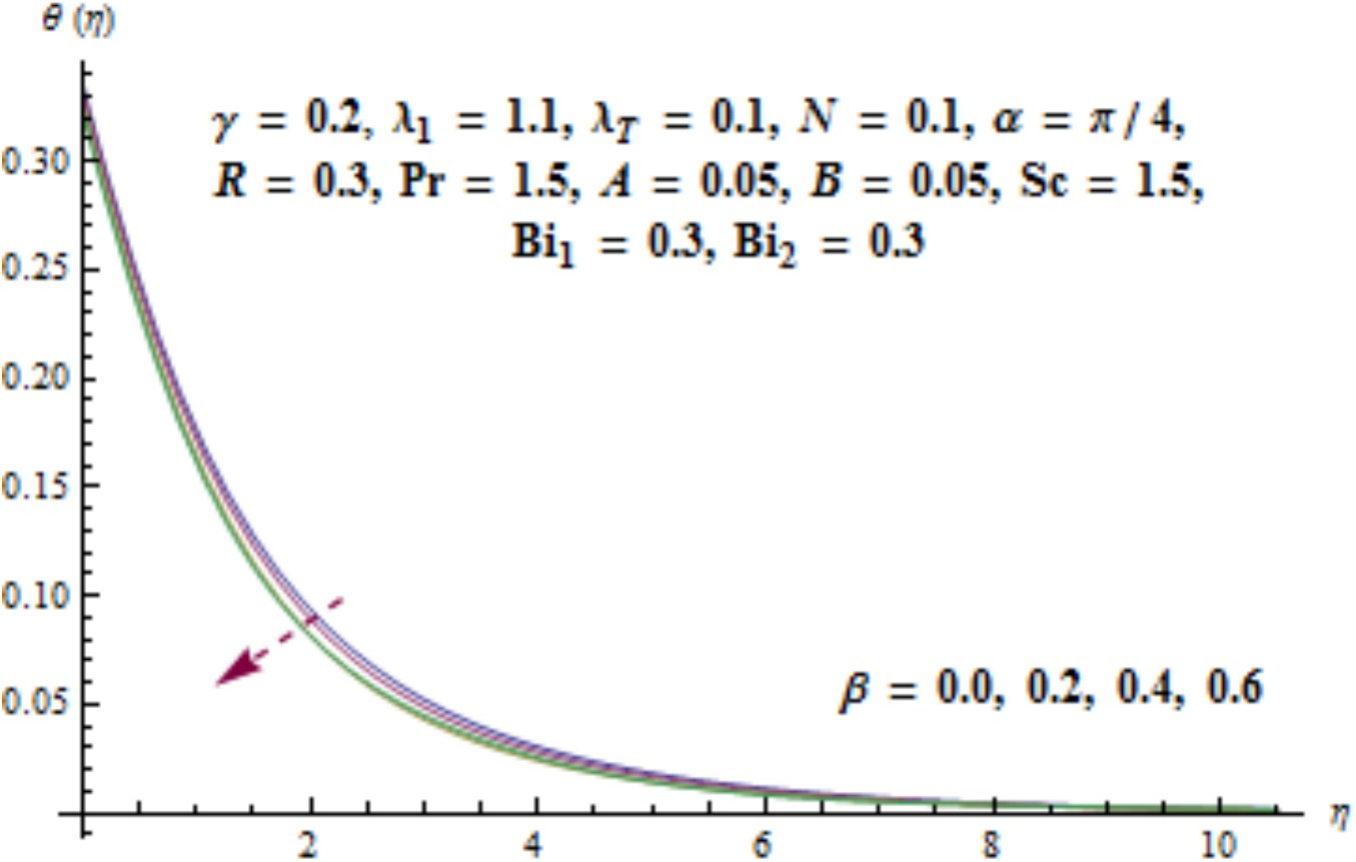
Behavior of *β* on *θ*(*η*).

**Fig 12:**
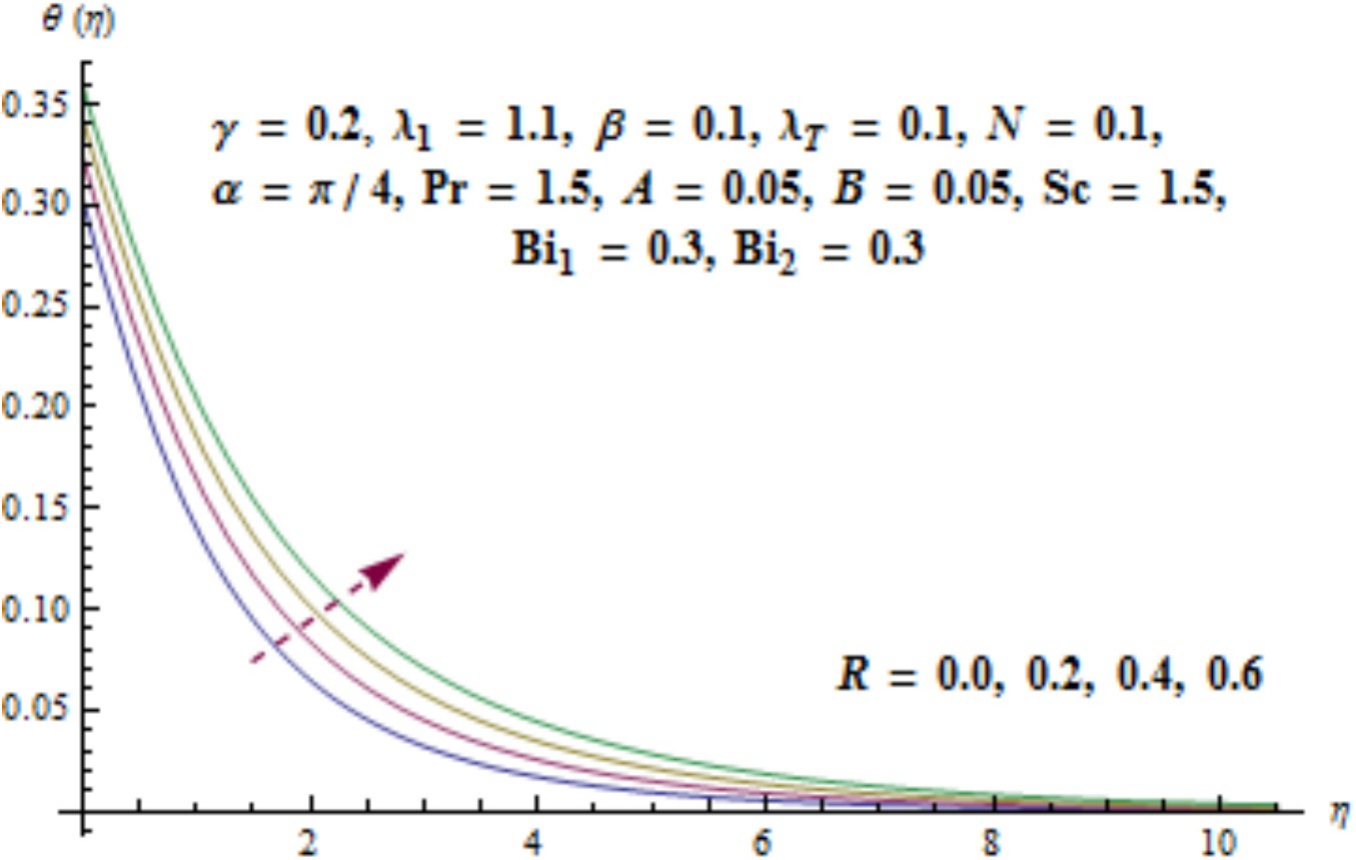
Behavior of *R* on *θ*(*η*).

**Fig 13:**
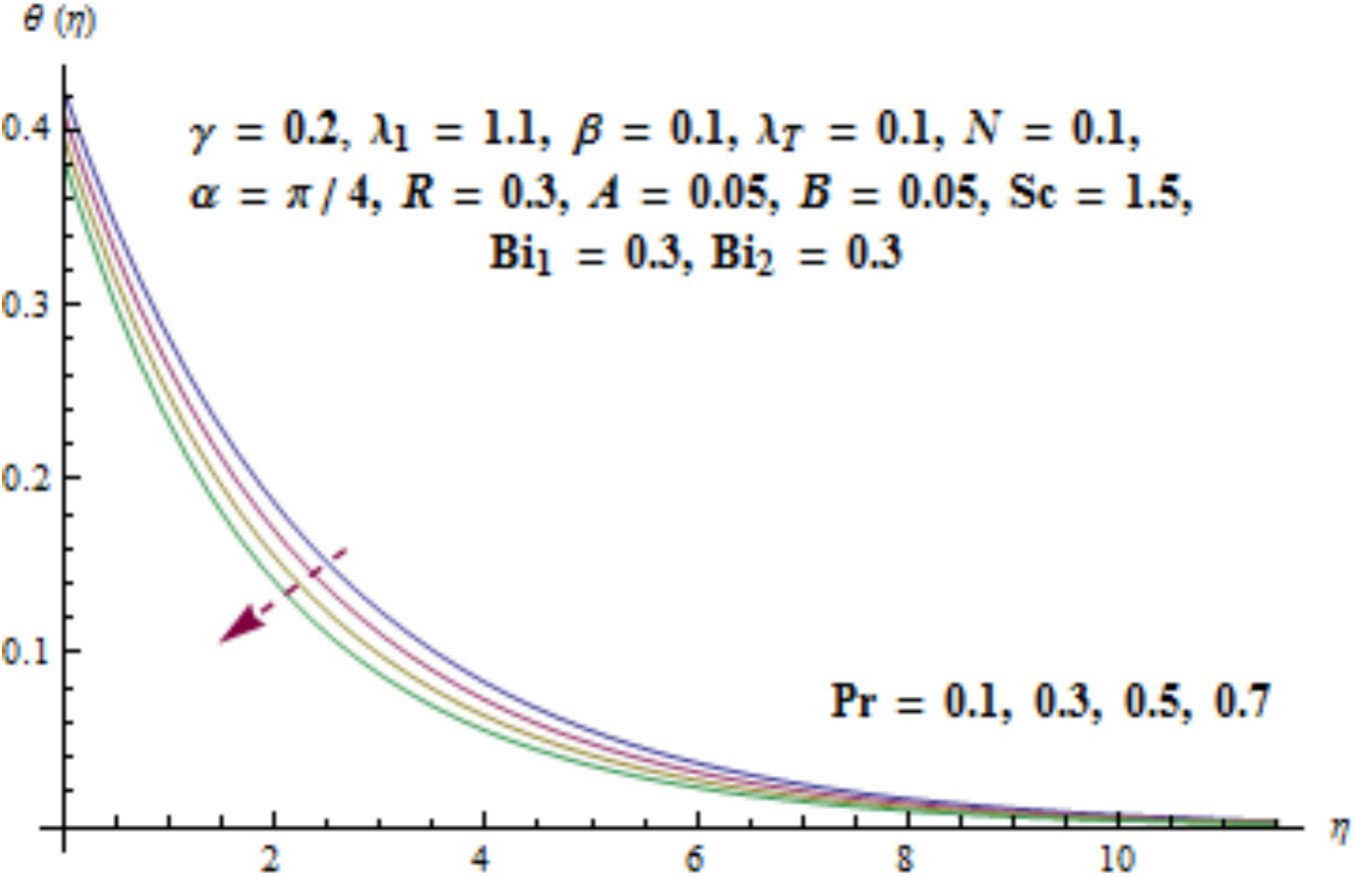
Behavior of Pr on *θ*(*η*).

**Fig 14:**
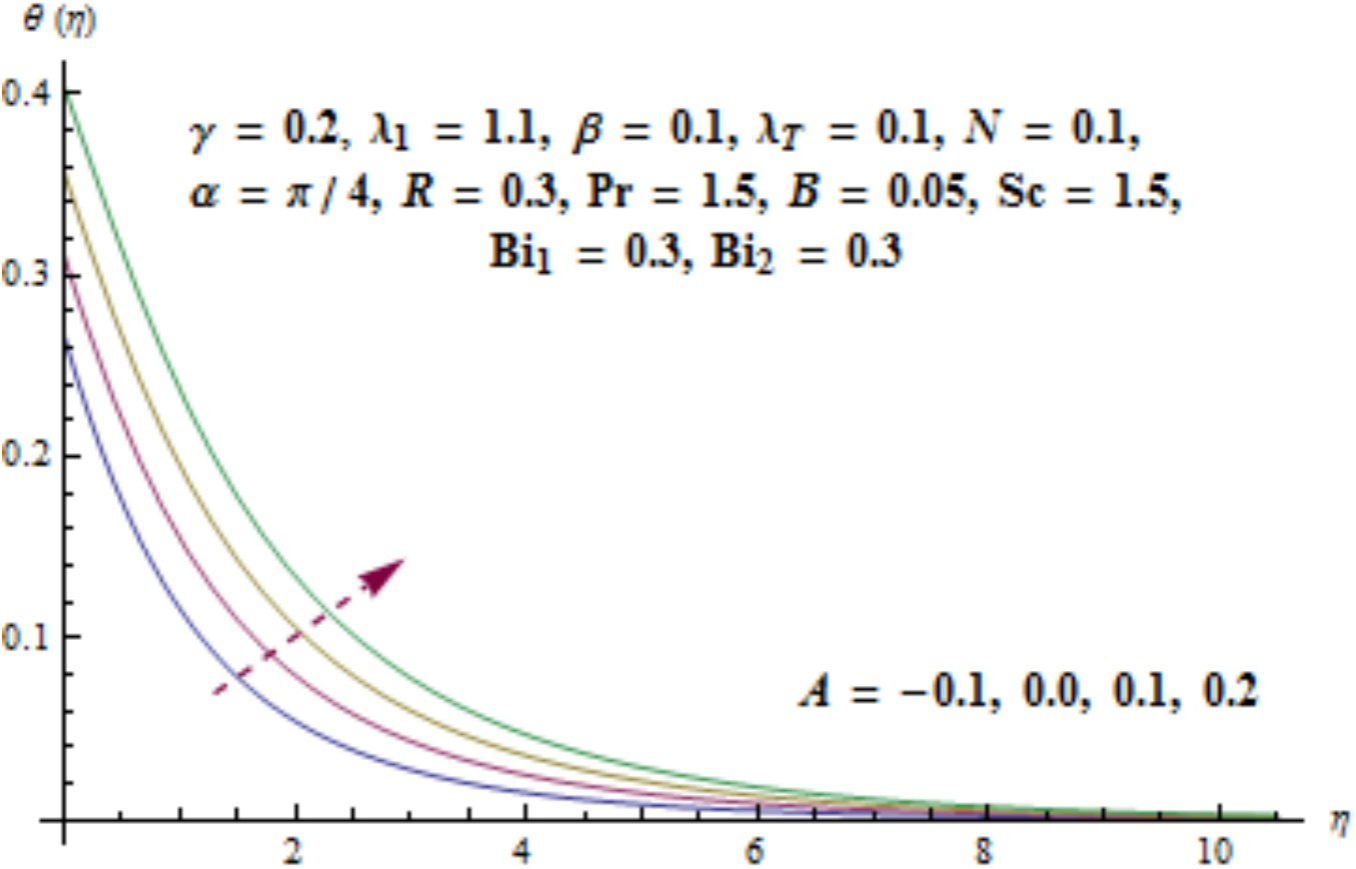
Behavior of *A* on *θ*(*η*).

**Fig 15:**
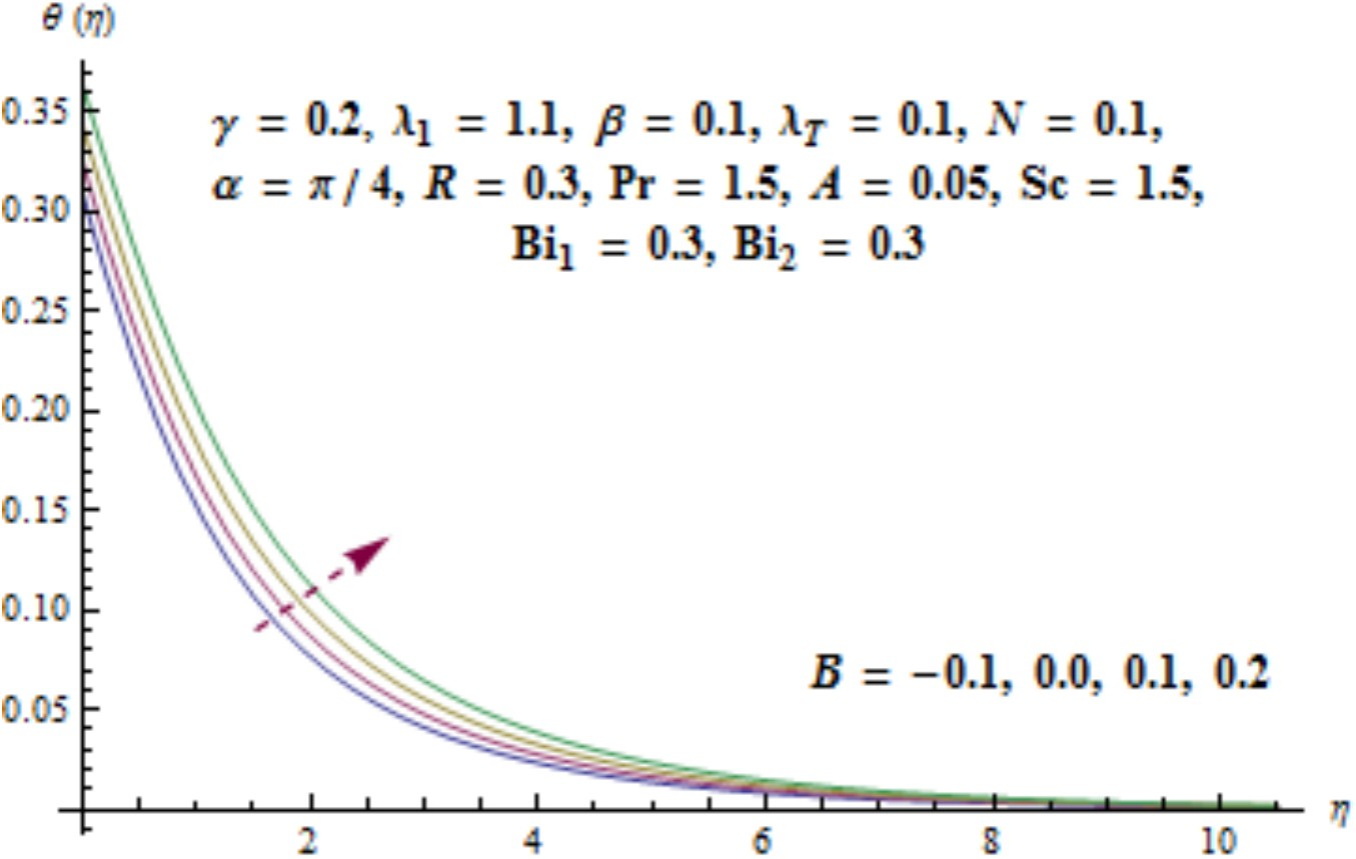
Behavior of *B* on *θ*(*η*).

**Fig 16:**
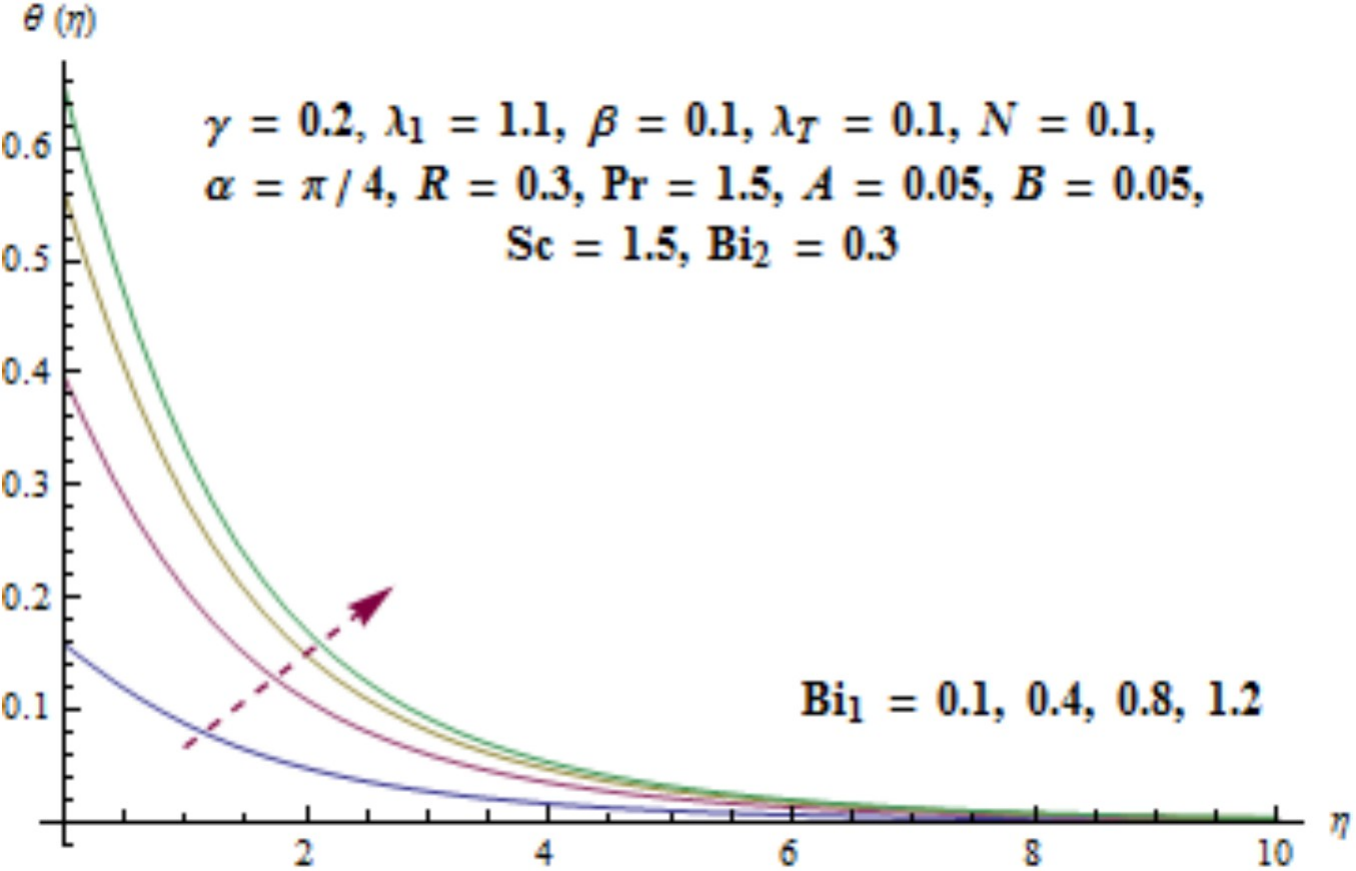
Behavior of *Bi*_1_ on *θ*(*η*).

Variation of curvature parameter *γ* on temperature distribution is expressed in Fig 9. Temperature has decreasing effect near surface of cylinder and it has increasing behavior far away from it. Fig 10 presents the impact of *λ*_1_ on temperature. Here we see that temperature field enhances for larger values of *λ*_1_. Since *λ*_1_ is the relaxation to retardation times ratio so for higher values of *λ*_1_ the relaxation time increases. It produces heat due to additional resistance to the fluid motion. Hence temperature distribution increases. Impact of Deborah number *β* on the temperature profile is shown in Fig 11. Here both temperature and thickness of thermal boundary layer decay for higher Deborah number *β*. Influence of thermal radiation *R* on temperature distribution is examined in Fig 12. It is found that temperature and thickness of thermal layer have increasing behavior for thermal radiation *R*. Physically it is verified as heat is produced due to radiation process in the working fluid so temperature field enhances. For various values of Prandtl number Pr the temperature is depicted in Fig 13. Here temperature and thermal layer thickness diminished when Prandtl number Pr enhances. It is due to the fact that larger Prandtl number Pr correspond to lower thermal diffusivity which results in the reduction of temperature distribution. Figs 14 and 15 describe the impacts of parameters *A* and *B* on the temperature distribution. It is analyzed that temperature and thickness of thermal boundary layer increase with larger values of heat (*A* > 0, *B* > 0). It is because of the fact that more heat is delivered due to heat source which is responsible in the enhancement of temperature. Fig 16 provides the analysis for various values of Biot number *Bi*_1_ on temperature distribution. Both temperature and thickness of thermal boundary layer have increasing behavior of thermal Biot number *Bi*_1_. In fact Biot number involves the heat transfer coefficient *h*_*t*_ so with the increase of *h*_*t*_ thermal Biot number enhances. Thus higher values of heat transfer coefficient lead to enhancement of temperature and thickness of thermal boundary layer.

**Fig 17:**
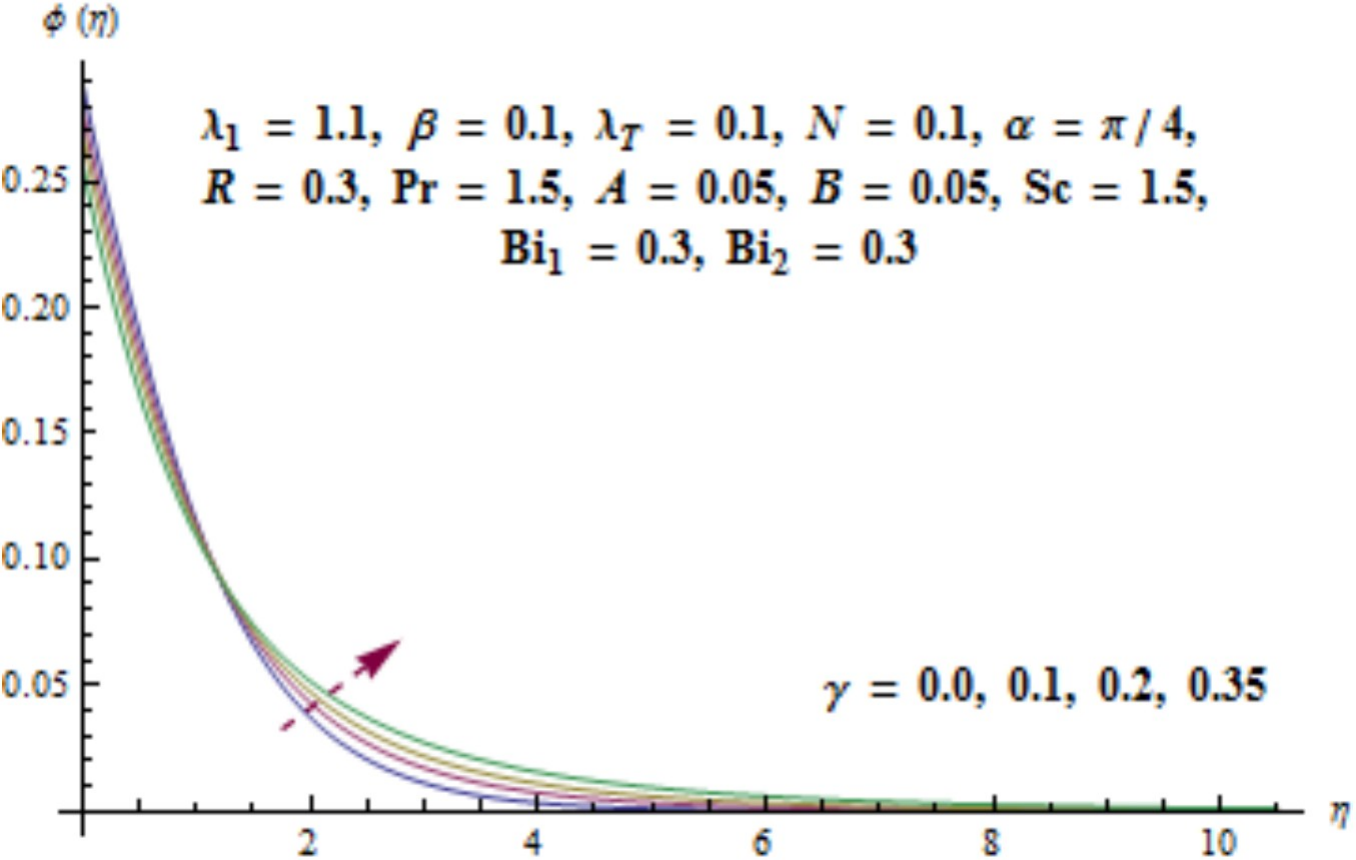
Behavior of *γ* on *ϕ*(*η*).

**Fig 18:**
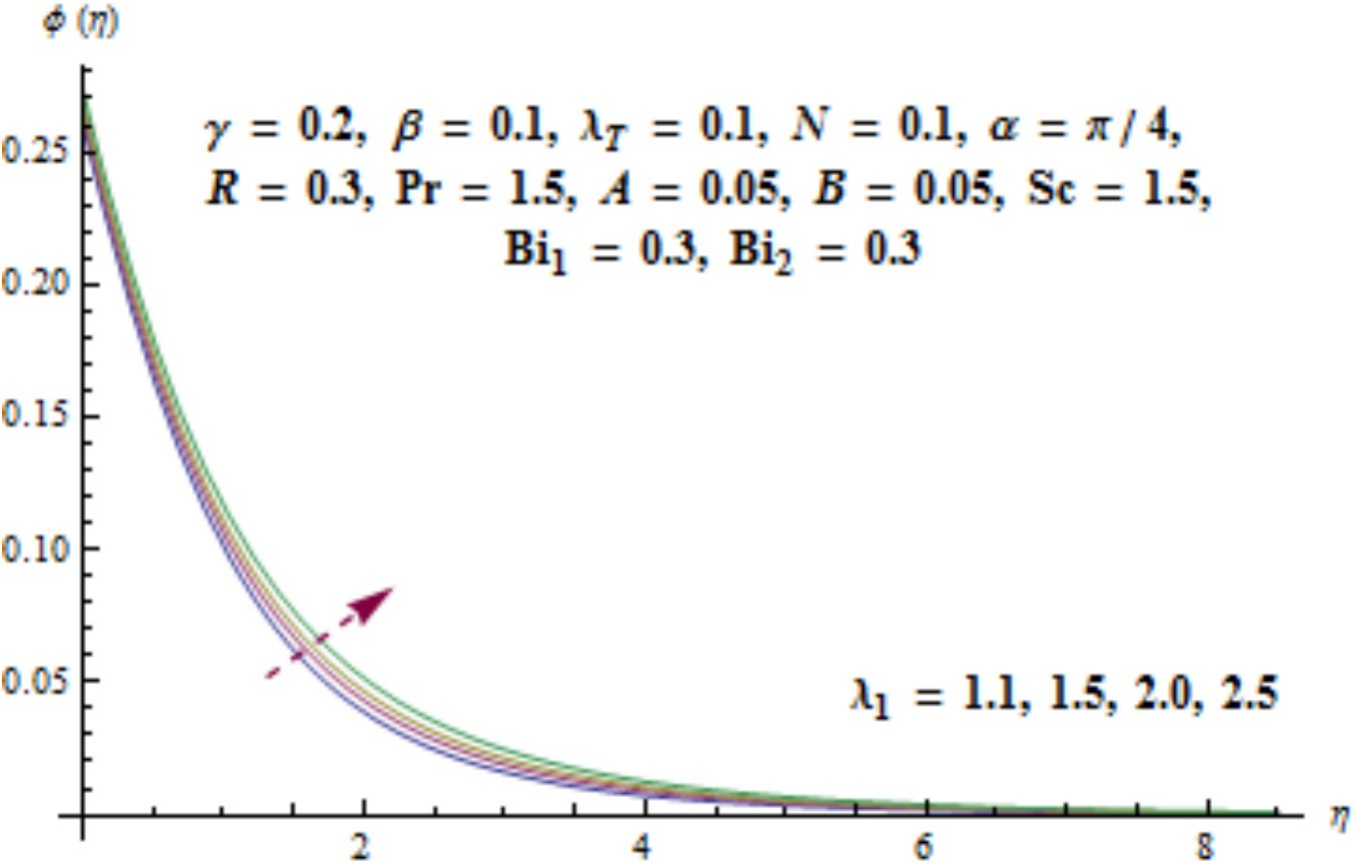
Behavior of *λ*_1_ on *ϕ*(*η*).

**Fig 19:**
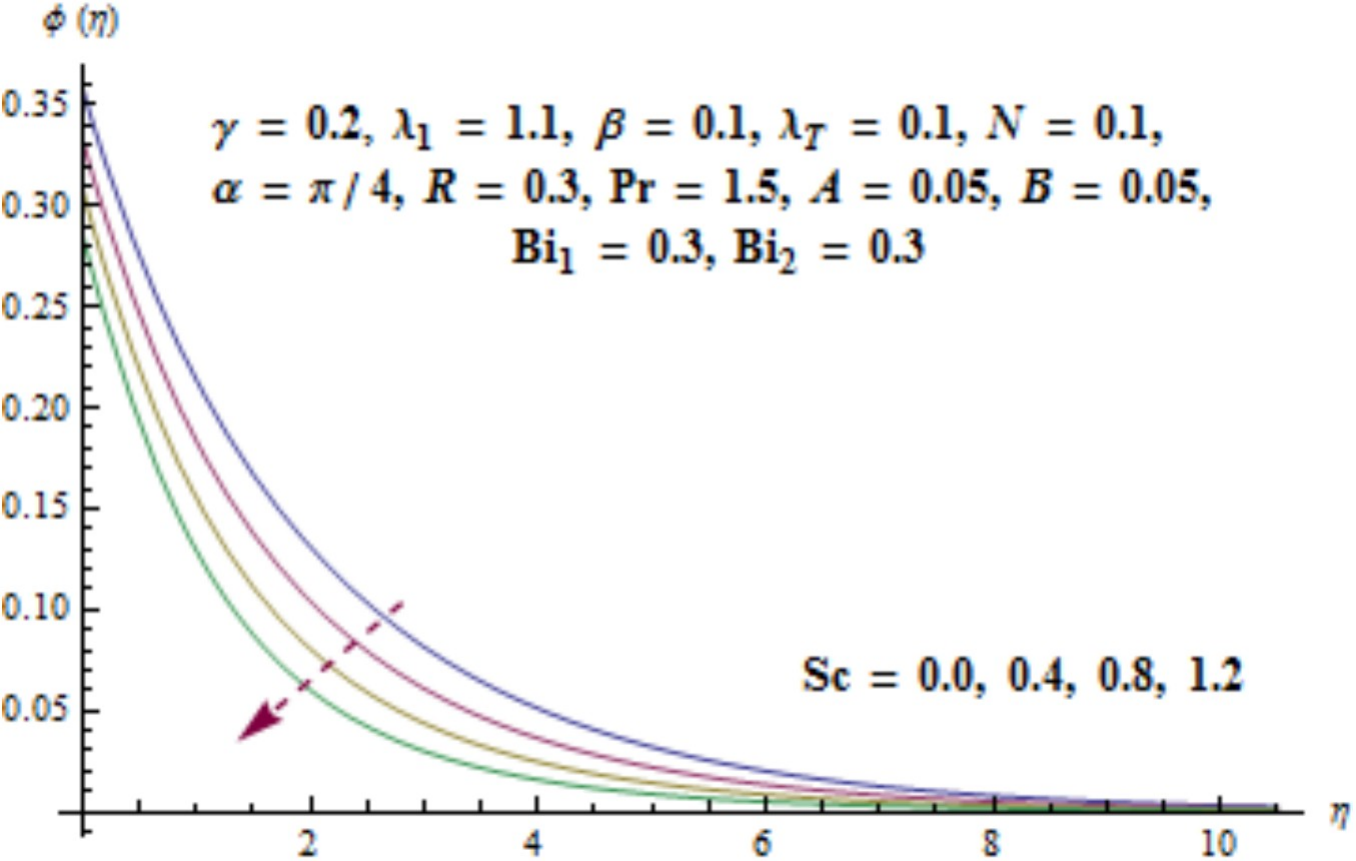
Behavior of *Sc* on *ϕ*(*η*).

**Fig 20:**
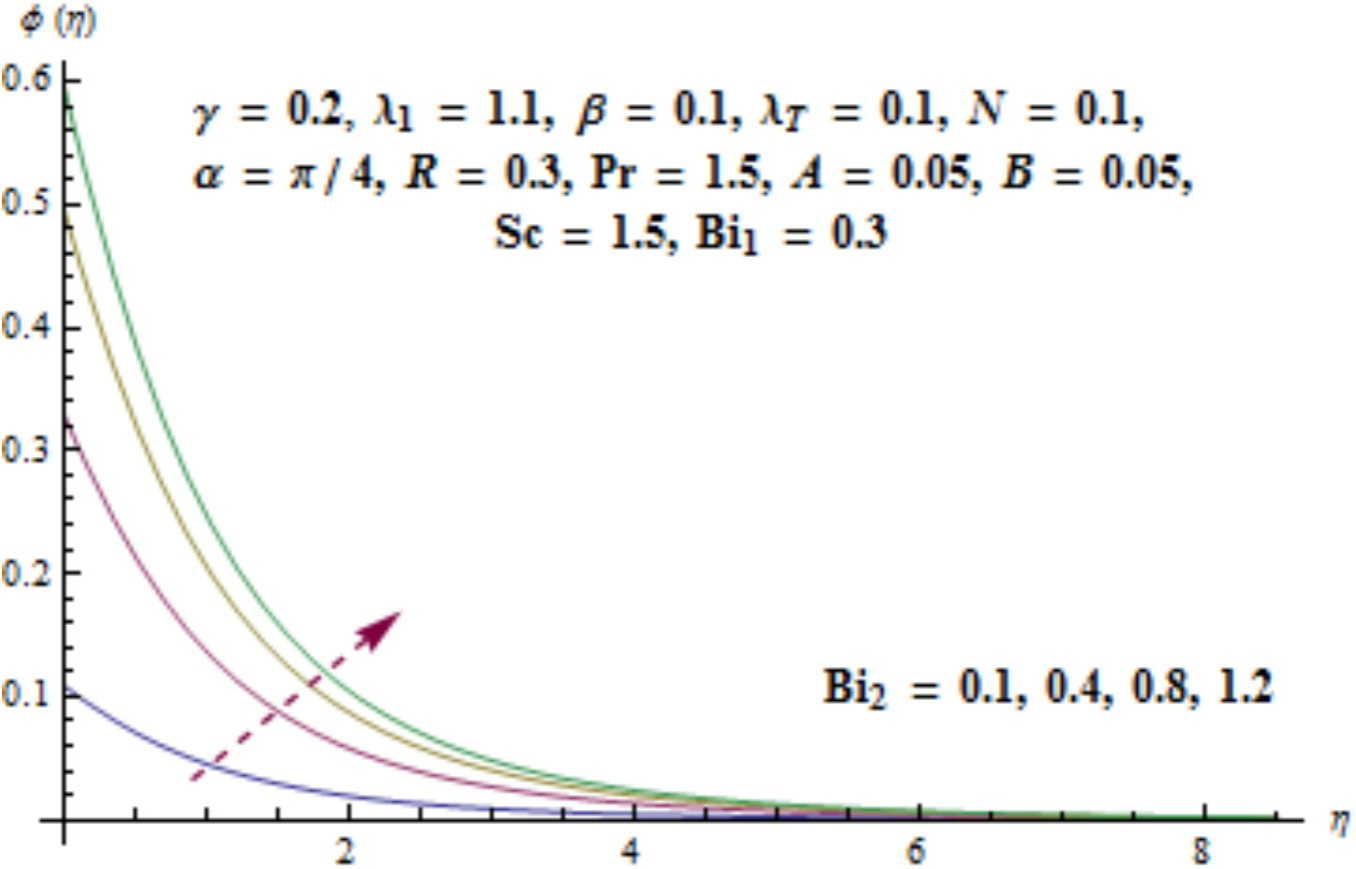
Behavior of *Bi*_2_ on *ϕ*(*η*).

Fig 17 illustrates that concentration distribution reduces near the cylinder and it enhances far away for higher values of curvature parameter *γ*. Fig 18 depicts the influence of *λ*_1_ on concentration. Higher values of *λ*_1_ results in the enhancement of concentration field and thickness of associated boundary layer. Characteristics of Schmidt number *Sc* on the concentration are captured in Fig 19. Concentration field reduces for higher values of Schmidt number *Sc*. Larger values of Schmidt number *Sc* lead to lower mass diffusivity which results in the reduction of concentration field. Analysis of mass Biot number *Bi*_2_ on the concentration is sketched in Fig 20. Obviously the concentration and thickness of associated boundary layer have increasing behavior for mass Biot number *Bi*_2_.

Table 1 demonstrates the convergence of homotopic solutions for the velocity, temperature and concentration. It is examined that 13^*th*^ order of approximation is enough for the convergence of velocity and 18^*th*^ order of approximation is enough for the convergence of temperature and concentration. Table 2 explores the behavior of various physical quantities on the skin friction coefficient. Here we observed that for increasing values of curvature parameter *γ*, Deborah number *β* and angle of inclination *α* the skin friction coefficient enhances while it shows decreasing behavior for larger relaxation to retardation times ratio *λ*_1_, mixed convection parameter *λ*_*T*_, ratio of concentration to thermal buoyancy forces *N* and Biot number *Bi*_1_. Behavior of various quantities on Nusselt number is explored in Table 3. It is examined that local Nusselt number is increasing function of curvature parameter *γ*, mixed convection parameter *λ*_*T*_, radiation parameter *R*, Prandtl number Pr, Deborah number *β* and Biot number *Bi*_1_. Nusselt number decreases when non-uniform heat source/sink parameter (*A* > 0 and *B* > 0) is increased. Table 4 is constructed to examine the behavior of various quantities on Sherwood number. It is noted that Sherwood number enhances for larger curvature parameter *γ*, Deborah number *β*, mixed convection parameter *λ*_*T*_, Schmidt number *Sc* and Biot number *Bi*_2_ while it decreases with increasing values of relaxation to retardation times ratio *λ*_1_ and angle of inclination *α*.

**Table 1:** Convergence analysis of the homotopic solutions for different order of approximations when *γ* = 0.2, *λ*_1_ = 1.1, *β* = 0.1, *λ*_*T*_ = 0.1, *N* = 1.0, *α* = *π*/4, *R* = 0.3, Pr = 1.5, *A* = 0.05, *B* = 0.05, *Sc* = 1.5, *Bi*_1_ = 0.3 and *Bi*_2_ = 0.3.

Order of approximation	−*f*″(0)	−*θ*′(0)	−*ϕ*′(0)
1	1.1587	0.20952	0.22518
5	1.3800	0.19306	0.21730
10	1.4174	0.18738	0.21482
13	1.4198	0.18594	0.21443
18	1.4198	0.18469	0.21427
25	1.4198	0.18469	0.21427
30	1.4198	0.18469	0.21427
35	1.4198	0.18469	0.21427

**Table 2:** Behavior of different physical quantities on skin friction coefficient when *R* = 0.3, Pr = 1.5, *A* = 0.05, *B* = 0.05, *Sc* = 1.5 and *Bi*_2_ = 0.3.

Parameters (fixed values)	Parameters		$-\frac{1}{2}Re_{x}^{0.5}C_{f_{x}}$
*λ*_1_ = 1.1,*β* = 0.1,*λ*_*T*_ = 0.1,*N* = 0.1,*α* = *π*/4,*Bi*_1_ = 0.3	*γ*	0.0	0.7082
		0.2	0.7469
		0.5	0.8031
*γ* = 0.2,*β* = 0.1,*λ*_*T*_ = 0.1,*N* = 0.1,*α* = *π*/4,*Bi*_1_ = 0.3	*λ*_1_	1.1	0.7469
		1.2	0.7285
		1.5	0.6806
*γ* = 0.2,*λ*_1_ = 0.1,*λ*_*T*_ = 0.1,*N* = 0.1,*α* = *π*/4,*Bi*_1_ = 0.3	*β*	0.1	0.7469
		0.2	0.7826
		0.5	0.8822
*γ* = 0.2,*λ*_1_ = 0.1,*β* = 0.1,*N* = 0.1,*α* = *π*/4,*Bi*_1_ = 0.3	*λ*_*T*_	0.1	0.7469
		0.2	0.7332
		0.5	0.6964
*γ* = 0.2,*λ*_1_ = 0.1,*β* = 0.1,*λ*_*T*_ = 0.1,*α* = *π*/4,*Bi*_1_ = 0.3	*N*	0.1	0.7469
		0.2	0.7460
		0.5	0.7432
*γ* = 0.2,*λ*_1_ = 0.1,*β* = 0.1,*λ*_*T*_ = 0.1,*N* = 0.1,*Bi*_1_ = 0.3	*α*	0.0	0.7411
		*π*/4	0.7469
		*π*/3	0.7511
*γ* = 0.2,*λ*_1_ = 0.1,*β* = 0.1,*λ*_*T*_ = 0.1,*N* = 0.1,*α* = *π*/4	*Bi*_1_	0.3	0.7469
		0.5	0.7424
		0.7	0.7393

**Table 3:** Behavior of various physical quantities on the local Nusselt number when *λ*_1_ = 1.1, *N* = 0.1, *α* = *π*/4, *Sc* = 1.5 and *Bi*_2_ = 0.3.

Parameters (fixed values)	Parameters		−Rex−0.5Nux
*β* = 0.1,*λ*_*T*_ = 0.1,*R* = 0.3,Pr = 1.5,*A* = 0.05,*B* = 0.05,*Bi*_1_ = 0.3	*γ*	0.0	0.2512
		0.2	0.2583
		0.5	0.2774
*γ* = 0.1,*λ*_*T*_ = 0.1,*R* = 0.3,Pr = 1.5,*A* = 0.05,*B* = 0.05,*Bi*_1_ = 0.3	*β*	0.1	0.2583
		0.2	0.2601
		0.5	0.2661
*γ* = 0.1,*β* = 0.1,*R* = 0.3,Pr = 1.5,*A* = 0.05,*B* = 0.05,*Bi*_1_ = 0.3	*λ*_*T*_	0.1	0.2583
		0.2	0.2600
		0.5	0.2641
*γ* = 0.1,*β* = 0.1,*λ*_*T*_ = 0.1,Pr = 1.5,*A* = 0.05,*B* = 0.05,*Bi*_1_ = 0.3	*R*	0.0	0.1988
		0.3	0.2583
		0.5	0.2953
*γ* = 0.1,*β* = 0.1,*λ*_*T*_ = 0.1,*R* = 0.3,*A* = 0.05,*B* = 0.05,*Bi*_1_ = 0.3	Pr	0.8	0.2021
		1.0	0.2225
		1.5	0.2583
*γ* = 0.1,*β* = 0.1,*λ*_*T*_ = 0.1,*R* = 0.3,Pr = 1.5,*B* = 0.05,*Bi*_1_ = 0.3	*A*	0.05	0.2583
		0.1	0.2443
		0.2	0.2144
*γ* = 0.1,*β* = 0.1,*λ*_*T*_ = 0.1,*R* = 0.3,Pr = 1.5,*A* = 0.05,*Bi*_1_ = 0.3	*B*	0.05	0.2583
		0.1	0.2443
		0.2	0.1862
*γ* = 0.1,*β* = 0.1,*λ*_*T*_ = 0.1,*R* = 0.3,Pr = 1.5,*A* = 0.05,*B* = 0.0.5	*Bi*_1_	0.3	0.2583
		0.5	0.3496
		0.7	0.4125

**Table 4:** Behavior of different physical quantities on the local Sherwood number when *N* = 0.1, *R* = 0.3, Pr = 1.5, *A* = 0.05, *B* = 0.05 and *Bi*_1_ = 0.3.

Parameters (fixed values)	Parameters		−Rex−0.5Shx
*λ*_1_ = 1.1,*β* = 0.1,*λ*_*T*_ = 0.1,*α* = *π*/4,*Sc* = 1.5,*Bi*_2_ = 0.3	*γ*	0.1	0.2119
		0.2	0.2146
		0.5	0.2215
*γ* = 0.2,*β* = 0.1,*λ*_*T*_ = 0.1,*α* = *π*/4,*Sc* = 1.5,*Bi*_2_ = 0.3	*λ*_1_	1.1	0.2146
		1.2	0.2141
		1.5	0.2127
*γ* = 0.2,*λ*_1_ = 0.1,*λ*_*T*_ = 0.1,*α* = *π*/4,*Sc* = 1.5,*Bi*_2_ = 0.3	*β*	0.1	0.2146
		0.2	0.2155
		0.5	0.2174
*γ* = 0.2,*λ*_1_ = 0.1,*β* = 0.1,*α* = *π*/4,*Sc* = 1.5,*Bi*_2_ = 0.3	*λ*_*T*_	0.1	0.2146
		0.2	0.2154
		0.5	0.2170
*γ* = 0.2,*λ*_1_ = 0.1,*β* = 0.1,*λ*_*T*_ = 0.1,*Sc* = 1.5,*Bi*_2_ = 0.3	*α*	0.0	0.2149
		*π*/4	0.2146
		*π*/3	0.2143
*γ* = 0.2,*λ*_1_ = 0.1,*β* = 0.1,*λ*_*T*_ = 0.1,*α* = *π*/4,*Bi*_2_ = 0.3	*Sc*	0.8	0.1888
		1.0	0.1985
		1.5	0.2146
*γ* = 0.2,*λ*_1_ = 0.1,*β* = 0.1,*λ*_*T*_ = 0.1,*α* = *π*/4,*Sc* = 1.5	*Bi*_2_	0.3	0.2146
		0.5	0.3004
		0.7	0.3626

## 4 Conclusions

Behaviors of non-uniform heat source/sink and thermal radiation in mixed convection flow of Jeffrey fluid due to an inclined stretching cylinder with convective conditions are addressed. We have see that velocity, temperature and concentration far away from the cylinder increase for larger curvature parameter. Larger non-uniform heat source/sink parameter (*A* > 0 and *B* > 0) lead to an enhancement in temperature field. The temperature and concentration increases for larger biot numbers. Larger Schmidt number *Sc* result in the reduction of concentration field. Larger values of heat source (*A* > 0 and *B* > 0) result in the enhancement of temperature profile. However Nusselt number decreases in this case. Variation of mixed convection (thermal buoyancy) parameter *λ*_*T*_ results in the enhancement of velocity field while skin friction coefficient decays. The presence of curvarure parameter leads to an enhance the skin friction coefficient, local Nusselt and Sherwood numbers.
